# A Systematic Review and Meta-analysis of the Effect of Active Video Games on Postural Balance

**DOI:** 10.1016/j.apmr.2023.01.002

**Published:** 2023-01-18

**Authors:** Caio Victor Sousa, Kelly Lee, Dar Alon, Dagmar Sternad, Amy S. Lu

**Affiliations:** a Health and Human Sciences, Frank R. Seaver College of Science and Engineering, Loyola Marymount University, Los Angeles, CA; b Health Technology Lab, Departments of Communication Studies and Health Sciences, College of Arts, Media, and Design, Bouvé College of Health Sciences, Northeastern University, Boston, MA; c Departments of Biology, Electrical & Computer Engineering, and Physics, Northeastern University, Boston, MA

**Keywords:** Active-video gaming, Exergaming, Meta-analysis, Postural balance, Posture sway, Rehabilitation, Systematic review, Telerehabilitation, Video games, Virtual reality, Virtual reality exercise

## Abstract

**Objective:**

To conduct a comprehensive systematic review and meta-analysis of the effects of active video game (AVG) interventions on postural balance across all ages in populations with and without neurologic impairments, using all types of platforms.

**Data Source:**

Six databases (PubMed, PsycINFO, Sport Discus, MEDLINE, Web of Science, and Google Scholar) were reviewed by December 31, 2020.

**Study Selection:**

The protocol was registered in the International Prospective Register of Systematic Reviews (PROSPERO: CRD42020204191). For inclusion, a study must be original, published in English peer-reviewed venues and employed AVGs as the sole or primary intervention to enhance, maintain, or regain postural balance. At least 2 within- or between-subjects conditions must be included with ≥10 participants per condition.

**Data Extraction:**

Three reviewers independently performed data extraction and assessed the risk of bias.

**Data Synthesis:**

129 studies were identified, with 102 eligible for meta-analysis. The total number of tested participants was 6407 (60.0% women, M_age_=55.1 years, range=3-99 years, SD=22.6). The average intervention duration was 35.6 min/session with 3.1 sessions/week for 7.6 weeks. The overall effect favored AVG interventions (Hedges’ *g*=0.469; 95% confidence interval [CI]=0.407-0.531). Although the overall study quality was relatively low, the analysis expectedly indicated significantly larger effects (*P*<.001) for AVG-interventions over passive controls (Hedges’ *g*=0.627; 95% CI=0.466-0.788), but importantly also favored AVG-interventions over conventional treatment (Hedges’ *g*=0.389; 95% CI=0.311-0.468). All clinical populations responded positively, although with different effect sizes (*P*=.023). Children experienced larger treatment effects (Hedges’ *g*=0.550; 95% CI=0.336-0.764), closely followed by seniors (Hedges’ *g*=0.529; 95% CI=0.402-0.656). The largest intervention effect on balance improvements was seen in healthy people without a medical condition (Hedges’ *g*=0.609; 95% CI=0.465-0.753).

**Conclusions:**

AVGs can produce postural balance improvements and better postural maintenance. All populations could benefit from AVG interventions.

## Introduction

Video games have become an integral part of our lives. In 2020 in the U.S. alone, there were 214 million video game players with about 46 million players reporting disabilities.^1^ Aside from entertainment, active video games (AVGs) requiring physical movement have also become popular, with a strong potential to exert positive effects on overall wellness and various medical conditions (eg, cerebral palsy, multiple sclerosis, Parkinson’s disease).^2^ One of the first AVGs, *Dance Dance Revolution*, involves dancing on a force-measuring pad with prescribed step patterns.^3^ This and some of the ensuing AVG developments focus on whole-body movements that exercise overall coordination and postural balance. Rather than reinforcing a sedentary lifestyle—a perennial criticism of video games, these games can stimulate mobility and help train standing balance. Nintendo Wii and Microsoft Xbox Kinect have become widely used platforms in rehabilitation.^4^ Given the prevalence of AVG products in the contemporary mediascape and the widespread need for balance training for the global aging population, this systematic review and meta-analysis studied the effect of AVGs on postural balance to provide an up-to-date comprehensive evaluation of this domain.

The control of balance is integral to all activities of daily living.^5^ Balance, or postural stability, is not only expressed in upright standing or in a sitting posture, but is also intricately intertwined with all manual and locomotor actions. Balance has been defined as the ability to maintain the center of mass over the base of support during a quiet stance as well as when reacting to external stimuli.^6^ Maintaining balance is a complex sensorimotor process, involving the regulation of the configuration of the multi-limb body by integrating information from all sensory modalities. A good sense of balance is necessary to assure accurate movements of our upper extremities for everyday activities and, importantly, to prevent falls and thereby minimize the risk of injury.

Impaired balance control is detrimental to multiple aspects of daily life and functional independence in old age and disease.^7^ The ability to maintain postural balance is impaired in individuals affected by a wide range of neurologic conditions such as Parkinson’s disease, multiple sclerosis, and stroke. A cardinal symptom of Parkinson’s disease, impaired balance doubles the risk of falling compared with age peers.^8^ Among people with multiple sclerosis, 50%-70% of individuals experience at least 1 fall over a 6-month period and 30%-50% of these experience recurrent falls.^9,10^ Studies of individuals after stroke indicated that up to 73% of patients have fallen at least 1 time per year post-stroke.^11^ The ensuing cascade of reduced mobility, injuries, and hospitalization is the same for all populations with neurologic conditions.

But the risk of falls also increases with age alone and falling in the healthy elderly is a big concern for public health. For example, in the United States (US), each year there are about 36 million falls among older adults 65 years and above.^12^ Falls and associated injuries are 1 of the major causes of hospitalization among older adults,^12^ and unintentional falls led to over 42,000 deaths in 2020.^13^ Falls have caused a severe burden for the health care system with medical costs totaling to more than $50 billion. Naturally, public health concerns have been raised to identify ways to prevent falling and thereby prolong a healthy independent life.^14^

These summary statistics reflect the necessity to provide therapeutic or preventive programs to avoid or reduce falls.^15^ Although physical and occupational therapy can improve balance, the resources required for conventional therapy can be prohibitive. Regular therapy is not only expensive but also requires time, access to transportation and (frequently) caregivers to assist at visits. Hence, increasing opportunities for home training is of paramount importance. And yet, even if one knows that a regular routine is crucial, the motivation for carefully designed exercise sessions frequently dwindles.

AVGs can address this problem. AVGs are now available on virtually all commercially available game consoles and active virtual reality platforms, also known as AVR^16^ or virtual reality training.^17^ Together with specially created software and hardware, AVGs have the potential to be used as affordable and highly accessible exercise platforms to improve health outcomes across different populations. AVGs’ unique interactive features afford players both engagement and enjoyment that distract them from perceiving physical exertions and, hence, prolong training adherence.^18^ Especially during the self-isolation enforced by the COVID-19 pandemic, home-based exercises have become a part of the life of most people.^19^

The efficacy of AVGs on health outcomes has been synthesized in several previous meta-analyses and systematic reviews. However, these synthesis articles tended to focus on specific questions and subpopulations and hence have limited generalizability. Notably, while the average age of gamers ranges from 35 to 44 years,^20^ most studies were focused either on children or on elderly people. For example, Wu at al^21^ conducted a meta-analysis of 11 studies on children with cerebral palsy and reported a positive role of AVGs on the improvement of their balance. Taylor et al^22^ meta-analyzed 10 studies on older adults and found that AVGs improved measures of mobility and balance. Previous review studies also tended to examine participants either with or without certain impairments, preventing them from comparing the different effects found for different populations. For example, while Pope et al^23^ analyzed 14 studies of AVG effects on rehabilitative outcomes and differential effects in the young and the old, all of them were patients. Suleiman-Martos et al^24^ scrutinized 18 studies of independent community-dwelling older adults and reported positive effects on balance. Hocking et al^25^ focused on AVGs’ effect on children with developmental disabilities (eg, spastic and hemiplegic cerebral palsy) and found small to medium effects on balance. Given these more selective analyses, the present meta-analysis and systematic review will include studies across all ages and populations including clinical and healthy groups followed by some more focused analyses addressing specific clinically relevant questions.

Lastly, some previous studies tended to conflate or limit their definitions of AVG and AVG platforms. For example, while the labels AVG and exergame can be used interchangeably,^22,23,26,27^ other studies employed different terms, such as virtual reality^21^ or active computer gaming.^28^ This naming confusion led to identical studies reviewed under both AVG and exergame categorizations. Moving forward, the term “active video games” will serve as an umbrella term encompassing both AVGs and AVR. We will consider all active video gaming platforms using a comprehensive and mutually exclusive taxonomy to allow the maximum coverage.

## Methods

To provide an updated and comprehensive systematic review and meta-analysis, the research was performed in accordance with the PRISMA (Preferred Reporting Items for Systematic Reviews and Meta-analyses) guidelines.^29^ The protocol was registered in the International Prospective Register of Systematic Reviews (PROSPERO: CRD42020204191).

### General selection criteria

The inclusion criteria for choosing articles were (1) intervention studies must be original and published in peer-reviewed English language journals or full-length conference proceedings; (2) AVGs must be interactive and powered by electricity; (3) AVGs must require gross motor movements beyond mere finger movements with the goal of enhancing, maintaining, or regaining postural balance; (4) the interventions must have AVGs as the sole or primary form of treatment; (5) the intervention design includes at least 2 conditions, within- or between-subjects, with ≥10 participants per condition; and (6) the intervention assessed longer-term effects of AVG training (beyond a single session). Studies that included only 1 group pre-/post-test or 1 group post-test-only design were excluded. The control conditions included either conventional training/therapies or a passive group.

### Primary outcomes

The primary outcomes comprised a variety of balance-related measures, which ranged from questionnaires with self-reports to clinical scores, to sophisticated quantitative metrics obtained through posturography, which measures ground reaction forces via forceplates either in a research laboratory or by the exergame platforms. While questionnaires and standardized clinical tests only require paper and pencil and thus are relatively easy to administer, they remain relatively coarse-grained in their ability to differentiate levels of balance ability (eg, Berg Balance score). Another relatively easy class of evaluations comprises scores for static and dynamic balance embedded in a functional behavior that quantify performance with a stopwatch, tape measure, or related easily available tools. One such frequently used test is the timed Up and Go test (TUG) that measures the time to get up from a chair, walk 3 meters, and return to sit down.^30^ The results are then related to reference scores. Several broader tests with additional requirements such as cognitive dual tasks can also be evaluated with such readily available measures. If forceplates and algorithmic tools were available, detailed quantitative posturo-graphic metrics could be obtained. A summary statistic of the center of pressure (CoP) on the forceplate is typically collected while standing as still as possible. Sometimes, additional dynamic activities were included to evaluate the effect of more complex demands of dynamic balance on the CoP.

### Search procedure and materials

Between March 2020 and December 2020, we searched electronic databases for relevant studies, including PubMed, EBSCO (PsycINFO, Sport Discus, MEDLINE), Web of Science, and Google Scholar. In Phase I of our search, we selected all synthesis articles (types: review, narrative review, systematic review, meta-analysis, and synthesis of synthesis article) published by April 30, 2020, in the Google Scholar or PubMed databases. This first phase retrieved 195 studies published between 2009 and 2020. We extracted all the original articles from these synthesis papers. For a synthesis of these synthesis articles, we first extracted the separate synthesis articles and then extracted the individual original articles from each of these separate synthesis articles. The search in Phase I retrieved 219 synthesis articles of which 18 were eliminated as duplicates, leaving 201 for further analysis. From these, 3274 individual articles were extracted.

In Phase II, we searched for additional individual original studies published between January 1, 2016, and December 31, 2020, to ensure that all recent original studies, even if not included in the synthesis articles, were considered. This search used Google Scholar, PubMed, EBSCO (PsycINFO, Sport Discus, MEDLINE), and Web of Science. In both phases, references to all relevant articles were screened for further inclusions. In addition, multiple leading authors were contacted to obtain the latest studies published in their fields. This search in Phase II identified 1866 individual articles.

**Fig. 1:**
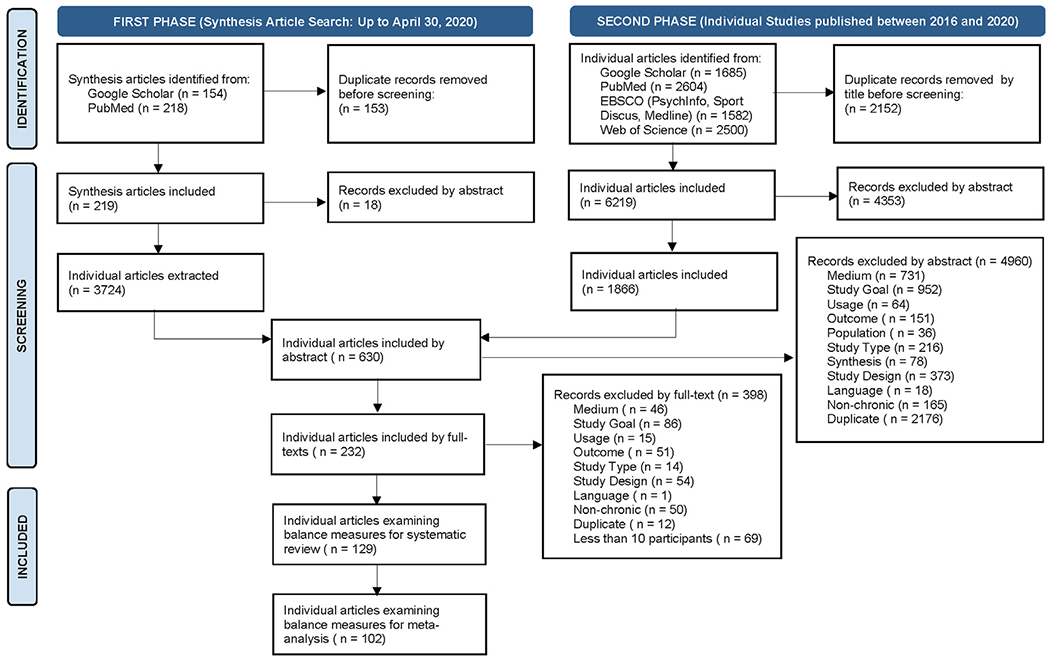
PRISMA 2020 flow diagram for the systematic review.

After pooling the 3274 and 1866 articles based on their abstracts, 630 unique articles were selected. After their full texts were read, 232 studies published between January 1996 and October 2020 were selected that fully met the inclusion criteria listed above. This 2-phase process proved more comprehensive than phase II alone, even without limiting the original publication’s time range. A summary of this 2-phase search process is displayed in Figure 1 (https://prisma-statement.org); the PRISMA checklist can be found in Table 1. The Boolean search phrases that were used for both Phases are listed in supplementary material 1.

### Data extraction and content coding

The articles were reviewed for eligibility by 3 independent coders (D.A., K.J.L., and C.V.S.) from different academic backgrounds (Biology, Health Science, and Physical Education) to provide a fair and comprehensive coverage. All coders had research experience in virtual games used for health purposes and were trained for 4 months in coding, using 5% of the included articles. During the preparation period (May to September 2020), the coders received 2 training sessions per week. An inter-rater reliability of >85% was achieved by assessing them repeatedly and randomly each week. For the remaining coding period, inter-rater reliability was maintained at >93%. Differences were resolved through discussion until all coders agreed on how to proceed.

Employing a standardized data extraction sheet, information about the characteristics of the study, its participants, the intervention, and the outcome measures were recorded for each selected article. Authors were contacted using a standardized email template when their publications did not contain all the information listed above.

### Evaluation of study quality

To assess the quality of the included articles, the Grading of Recommendations Assessment, Development and Evaluation (GRADE) assessment was implemented (Atkins, 2004). The 6 components of GRADE included (1) random assignment (to avoid selection bias), (2) allocation concealment (to avoid selection bias), (3) blinding of participants and personnel (to avoid performance bias), (4) blinding of outcome assessment (to avoid detection bias), (5) completeness of reporting of some outcome data (to avoid attrition bias), and (6) selectivity in reporting (to avoid reporting bias). We added a seventh criterion which assessed whether there was any potential adjustment for additional confounding variables. Each category was given a score from −1 to +1, where −1 represented high risk of bias, 0 represented unclear risk, and 1 represented low risk of bias. The overall score was an average of each individual component on the −1 to +1 scale. None of the articles were excluded based on their GRADE quality assessment.

### Statistical analysis

Statistical heterogeneity between studies was quantified using the *I*^2^ statistic, which describes the percentage of variation across studies due to heterogeneity rather than sampling error, or chance (0%-40%: negligible heterogeneity; 30%-60%: moderate heterogeneity; 50%-90%: substantial heterogeneity; 75%-100%: considerable heterogeneity).^31^ The level of significance was set at *P*<.05. Each outcome was combined and calculated using the MS Office Excel macro sheets provided by Borenstein et al.^32^ Statistical analyses were conducted with the software package Comprehensive Meta-Analysis^a^ (CMA 3.0, BioStat Inc, Englewood, NJ, USA).

### Meta-analytical procedure

For the meta-analysis, the study designs were homogenized by only using post-intervention measures. Parameters in the same category within a study were pooled with a fixed-effects model. Next, with sufficient homogeneity in design and comparators, a random-effects model was used for all meta-analyses. Continuous outcomes were converted into standardized mean differences (SMD) with a 95% confidence interval (CI). The SMD constituted the effect size measures for calculating Hedges’ *g*. This metric is generally preferred to Cohen’s *d* because it performs better when sample sizes are small but significantly different from each other. Our meta-analytical procedures followed methods outlined by Borenstein et al.^32^

An independent meta-analytical model was applied to each subgroup analysis. The subgroups were categorical moderators in each model and were defined based on the characteristics of the individual studies: “age-group” (eg, children, adults, seniors), “medical condition” (eg, cerebral palsy, Parkinson’s disease, stroke, etc), “type of control group” (eg, passive control, conventional therapy), “intervention length” (eg, short, medium, long), “platform development” (eg, Commercially Available, Special Development), “AVG platform” (eg, Wii, Xbox), and “intervention environment” (eg, laboratory, hospital, school). Additionally, we have included 5 analysis models with specific subgroups and moderators to address some relevant clinical questions. In order to determine the risk of bias, Egger’s test was applied, and the Funnel plot symmetry/asymmetry was assessed visually. The significance level for Egger’s regression was set at *P*<.1.^33^

## Results

### Samples and settings

After examining the outcomes of the 232 articles that met the general inclusion criteria, 129 studies were identified that reported 1 or more balance measure outcomes (see supplementary material 2 for each included study’s characteristics). Of those, 102 articles provided sufficient information to be eligible for the meta-analysis (eg, means, standard deviations, and sample sizes for the pre-post comparison). The first study was published in 2009^34^ and the number of studies increased over the 11 years until 2020.

### Participant details

The total enrollment across all 129 studies was 6407 participants (M_Total_=50±85.3, range=20-977; M_Experiment_=24±44.6, range=10-508; M_Control_=26±41.1; range=10-469). Although the average age of the participants was 55.1 years, the studies involved participants from a wide range of ages (range=3-99 years, SD=22.6): 12.4% studies involved children (0-18 years), 18.6% studies involved adults (18-64 years), 41.1% studies focused on seniors (>64 years), and 27.9% studies included both adults and seniors (>18 years). The average percentage of men participants across all studies was 40% (SD=19.8%; range=0%-100%).

Thirty-seven studies (28.7%) reported no specific medical conditions for the participants, that is, were conducted on healthy participants. The rest were conducted with clinical populations: stroke survivors (27, 20.9%), Parkinson’s disease (14, 10.9%), multiple sclerosis (8, 6.2%), cerebral palsy (7, 5.4%), diabetes (5, 3.9%), and several other conditions pooled into another group (31, 24%).

Seventy-eight (60.5%) studies were conducted in a field setting, with 9 (6.9%) performed in a laboratory setting; 31 (24.0%) studies did not specify the setting; and 11 studies were coded as other settings, for example, the study was conducted in more than 1 setting. Of the 78 field studies, most were conducted in physical therapy offices (31, 24.0%), followed by hospitals (20, 15.5%), homes (10, 7.8%), schools (9, 7.0%), or group living facilities (8, 6.2%).

The 129 studies were conducted around the world with their locations distributed across 6 regions: Asia (60, 47%), Europe (36, 28%), North America (14, 11%), South America (9, 7%), Oceania (7, 5%), and Africa (3, 2%). The top 3 countries were Taiwan (16, 12%), South Korea (14, 11%), and the US (11, 9%).

### Intervention characteristics

AVG training displayed a wide range of intervention durations and frequencies: from 10 to 100 minutes per session (M=35.6±15.3), from 1 to 7 sessions per week (M=3.1±1.3), and from 1 to 26 weeks (M=7.6±3.9). The average total intervention length was 920±706 minutes and ranged from 75 to 3900 minutes.

In terms of the AVG platforms, 21 (16.3%) were computer-based, for example, a computer game supported by a Kinect camera, and 87 (67.4%) required a commercially available gaming console. Among the consoles, Nintendo Wii or Wii U were the most popular (65, 50.4%), followed by the Microsoft Xbox Kinect (20, 15.5%). Fourteen (10.9%) required integrated AVG-specific equipment, such as a stationary bike or a dancing mat, while 7 (5.4%) did not specify the platform.

The 129 studies used a total of 413 AVGs as balance interventions. After excluding duplicate games, there were 230 unique games. None of them required an Internet connection for gameplay. In addition, AVGs were also coded for whether they used narratives for motivation.^35^ Surprisingly, none of the AVGs involved a narrative.

Most of the published balance interventions employed commercially available AVGs (95, 73.6%). Table Tilt (24), *Ski Slalom* (19), and *Penguin Slide* (18) were among the most frequently used games. Only 19 (14.7%) studies created AVGs through government funding or foundation grants for research and development. The remaining 15 (11.6%) studies did not report sufficient information to determine the source of funding for AVG creation.

### Risk of bias in individual studies

**Fig. 2:**
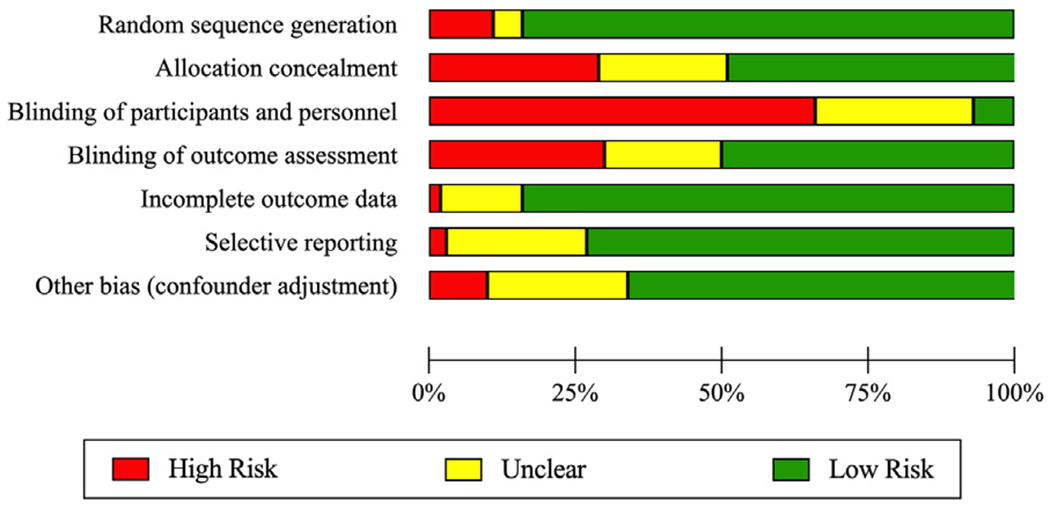
Study quality according to GRADE tool.

The overall study quality score, GRADE, was calculated by averaging the scores for the 7 components on a −1 to +1 scale (−1=low quality; +1=high quality). The distribution of GRADE scores on all 7 components is summarized in Figure 2. Overall, the mean GRADE average was low: −.38 (range: −1 to .43; SD = −.31). The top high-risk factor for bias was the lack of blinding of participants and personnel (67% articles). More than a quarter of the studies showed risk of bias due to allocation concealment and due to blinding of outcome assessment. Conversely, the top 4 factors indicating higher quality or low risk of bias were completeness in reporting outcome data (84%), random assignment (83%), avoiding selective reporting (75%), and other factors avoiding potential bias (66%).

The mean GRADE average was correlated with available information about the year of publication, study location, participant age, and intervention duration. The correlations were non-significant (*P*s.= .06 to .68), suggesting that the quality of the publication did not change over the years (2009-2020) and did not depend on the location, age, or intervention duration.

### Evaluation metrics

The outcome metrics that evaluated balance-related effects included a wide variety of scores and quantitative metrics. The reported results were grouped into 5 categories defined by the 5 categories of metrics.

The first category “Questionnaires/Clinical Scores” comprised measured scores of performance compared against normative data. A prominent example is the Berg Balance score that assigns integer values to 14 different activities involving static and dynamic balance. As stated previously, although these are relatively easy to administer, they are on an integer scale and therefore more coarse-grained than metrics on an analog scale.

A second class of metrics assessed functional actions involving static and dynamic balance that were evaluated with a stopwatch, a ruler, or a related easily available tool. As mentioned previously, the most prominent example is the TUG. Normative data exist for different geriatric or patient populations. This category was referred to as “Functional Balance”.

Several tests include additional tasks such as cognitive tasks to be carried out during the functional activities. Despite the more encompassing goal, these tests still rely on simple summary measures, such as time taken or distance reached that are compared with normative score tables. They are assigned to the category “Functional Balance+”.

“Posturography” was conducted if a study used forceplates and algorithmic tools to quantify the findings. The most frequently used assessment of static balance involved measuring the CoP by a forceplate. This sophisticated biomechanical signal is typically collected while standing as still as possible for 30 seconds, sometimes with a fixed gaze. The variability of the CoP or also center of mass due to potential balance impairments manifests in the fluctuations of the signal over time. These are summarized in standard deviations in the medio-lateral and anterior-posterior direction and other related statistical metrics.

The final category comprised the same type of posturography, but the variations in the CoP were measured during dynamic game activities (Posturography+). These tests often measured relatively complex dynamic balance via considerably higher fluctuations of the CoP.

A summary of the 5 types of balance outcomes can be found in Table 2.

### Meta-analyses

**Fig. 3:**
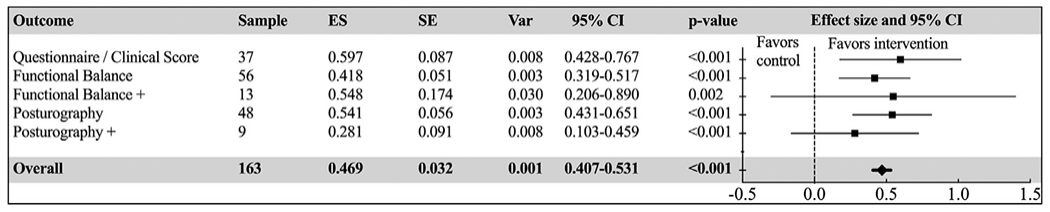
Forest plot of standardized mean effect sizes of the five categories of balance outcomes after AVG intervention. ES, effect size (Hedges’ *g*); SE, standard error; Var, variance.

A total of 163 different balance outcomes were reported in the 102 studies. “Functional Balance” was the most common category in 56 studies, followed by “Posturography” in 48 studies, and “Questionnaire/Clinical Scores” in 37 studies. The overall results showed a significant effect with positive results in favor of the intervention (Hedges’ *g*=0.469; 95% CI=0.407-0.531), with the largest effects documented in the category “Questionnaire/Clinical Scores” (Hedges’ *g*=0.597; 95% CI=0.428-0.767), closely followed by Functional Balance+ and Posturography (see Fig. 3). However, this analysis showed only a borderline between-group effect (*P*=.053) with moderate-to-substantial heterogeneity (*I*^2^ = 64.3%, Q=453.7); “Functional Balance+” was the most heterogeneous category (*I*^2^=82.1%, Q=66.9). Individual effects and supporting data are available as supplementary material 3.

**Fig. 4:**
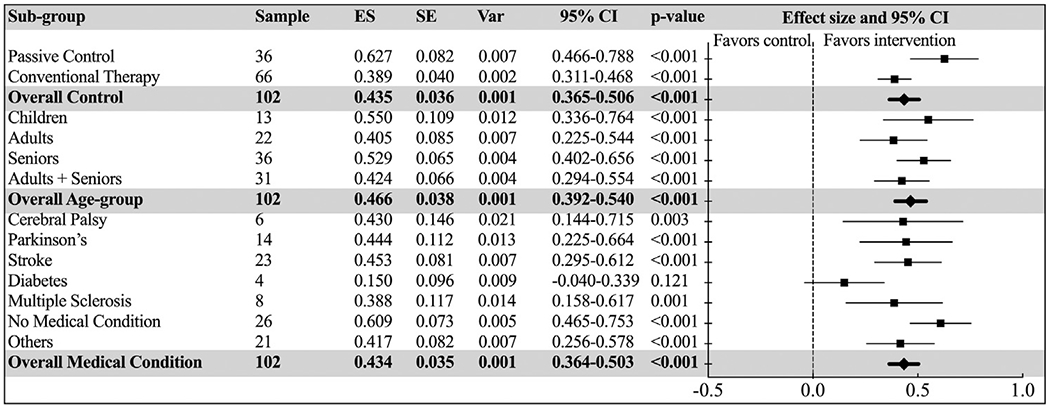
Forest plot of standardized mean effect sizes of subgroup analyses with “Type of Control Group”, “Age Group”, and “Medical Condition” as independent moderators. ES, effect size (Hedges’ *g*); SE, standard error; Var, variance.

Significant subgroup effects were found in all models (*P*s<0.001). A first comparison established that an AVG clinical intervention provided significant effects on balance when compared with a passive control group (*P*<.001), as to be expected (Hedges’ *g*=0.627; 95% CI=0.466-0.788). More interestingly, an AVG intervention also proved more effective than conventional therapy, although with a slightly smaller effect size (Hedges’ *g*=0.389; 95% CI=0.311-0.468). While the 3 age-groups did not show significant differences in their response (*P*=.404), children had a slightly larger effect (Hedges’ *g*=0.550; 95% CI=0.336-0.764), which was closely followed by seniors (Hedges’ *g*=0.529; 95% CI=0.402-0.656). Individuals with different medical conditions responded differently to the interventions (*P*=.023), with the largest effects in healthy people without a medical condition (Hedges’ *g*=0.609; 95% CI=0.465-0.753). Individuals with cerebral palsy, Parkinson disease, stroke, and multiple sclerosis all benefitted significantly from AVG interventions (*P*s≤0.003), while only people with diabetes did not (*P*=.121) (see Fig. 4).

While all intervention lengths rendered significantly different effects (*P*s < 0.001), there was also a significant difference (*P*=.018) among different intervention lengths. “Long Interventions” (longer than 8 weeks) showed the greatest improvements (Hedges’ *g*=0.561; 95% CI =0.386-0.736), and “Short Interventions” (less than 5 weeks) showed the smallest improvements (Hedges’ *g*=0.330; 95% CI=0.238-0.422).

There was no significant difference between the different platform development groups (*P*=.918), although most studies (81, 79.4%) used a commercially available platform for AVG interventions. No significant differences were observed between AVG platform models (*P*=.305), although XBox produced the largest intervention effect (Hedges’ *g*=0.636; 95% CI = 0.364-0.909), followed by “others”, for example, including more than 1 console, such as both Wii and Xbox, or Computer and Wii (Hedges’ *g*=0.622; 95% CI=0.280-0.964). Nintendo Wii was the most widely used platform (55, 53.9%).

**Fig. 5:**
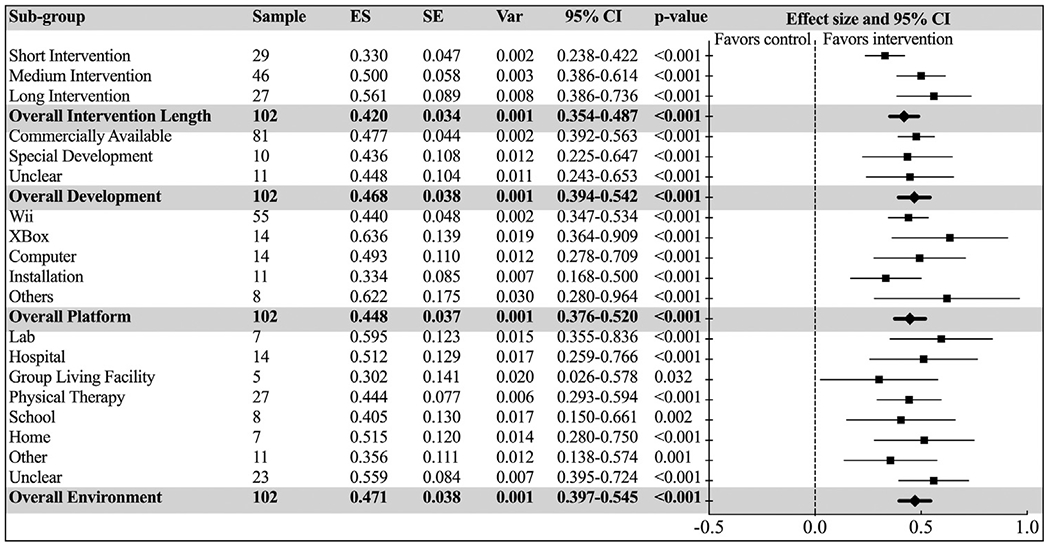
Forest plot of standardized mean effect sizes of subgroup analyses with “Intervention Length”, “Platform Development”, “Platform”, and “Environment” as independent moderators. ES, effect size (Hedges’ *g*); SE, standard error; Var, variance.

The last statistical model with all studies included indicated no significant differences between the environment subgroups (*P*=.629) although, not unexpectedly, studies conducted in the laboratory tended to elicit the largest effects (Hedges’ *g*=0.595; 95% CI=0.355-0.836). Physical therapy practice was the most common environmental subgroup (28, 27%) (see Fig. 5).

**Fig. 6:**
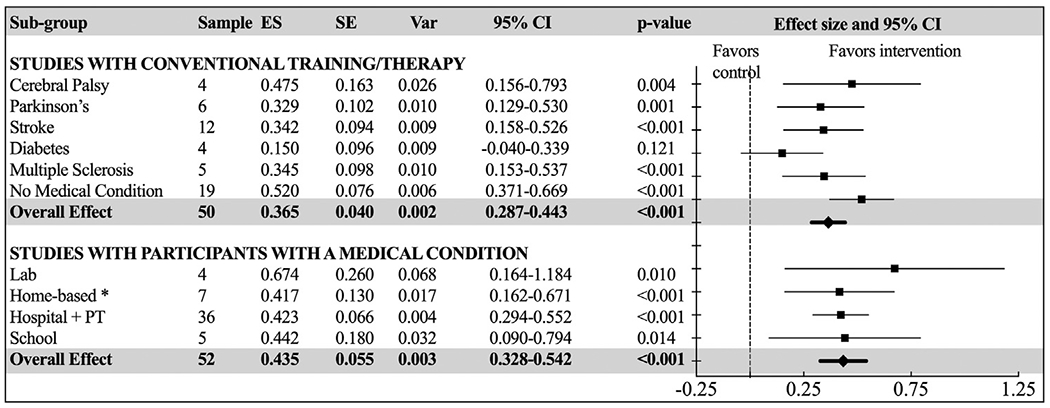
Forest plot of standardized mean effect sizes of focused subgroup analyses with “Medical Condition”, and “Environment” as independent moderators. ES, effect size (Hedges’ *g*); PT, physical therapy practice; SE, standard error; Var, variance; *Group living facilities.

To provide more focused answers to clinically relevant questions, 5 additional analyses were conducted on selected subgroups of studies. Model 1 focused on studies where the control group received conventional training/therapy, using participants’ medical condition as a moderator to provide more insights about the differential effects of AVG intervention. Results showed a borderline effect between the populations (*P*=.080). Similar to the overall model in Figure 4 that reported higher beneficial effects for the AVG intervention than passive control, this subgroup analysis confirmed that healthy subjects with no medical condition reaped the highest benefit from the AVG interventions (Hedges’ *g*=0.520; 95% CI = 0.371-0.669), and they were closely followed by individuals with cerebral palsy (see Fig. 6).

Model 2 included only studies with participants who had medical conditions with Environment as a moderator to explore whether the study setting would make a difference. Results did not detect significant differences between the intervention environments (*P*=.825), and only showed moderate effects across the board (*P*s<0.014) (see Fig. 6).

**Fig. 7:**
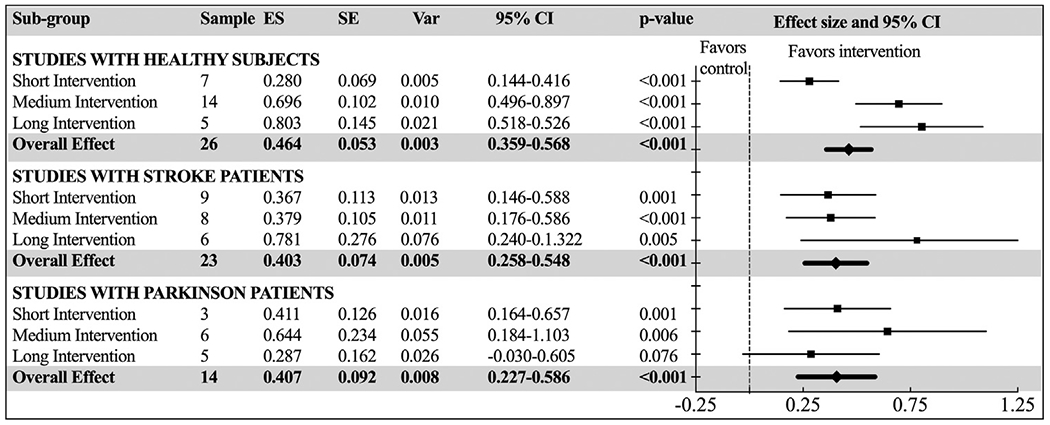
Forest plot of standardized mean effect sizes of focused subgroup analyses of healthy subjects, stroke patients, and Parkinson patients with “intervention length” as independent moderator. ES, effect size (Hedges’ *g*); SE, standard error; Var, variance.

Three additional subgroup analyses included studies with 1 of 3 participant subgroups, healthy subjects without any medical condition (Model 3), stroke patients (Model 4), and Parkinson’s disease patients (Model 5) regardless of the control group. The question was whether the duration of AVG intervention would make a difference for these 3 major populations. The overall effect indicated that all 3 subgroups significantly benefited from the AVG intervention (*P*s<0.001). Among healthy subjects (Model 3), there were significant differences between the different intervention lengths (**P**<.001), with the long interventions producing the highest effect size (Hedges’ *g*=0.803; 95% CI=0.518-0.526) and short interventions producing the lowest effect size (Hedges’ *g*=0.280; 95% CI=0.144-0.416). In contrast, among stroke patients (Model 4) and Parkinson’s disease patients (Model 5), there was no significant difference between the different intervention lengths (*P*=.363 and 0.457, respectively). Nevertheless, there was a trend showing that longer interventions produced higher effects for stroke patients. For Parkinson’s patients, though, medium intervention length seemed to produce the highest effects (see Fig. 7 for Models 3-5).

### Risk of publication bias

**Fig. 8:**
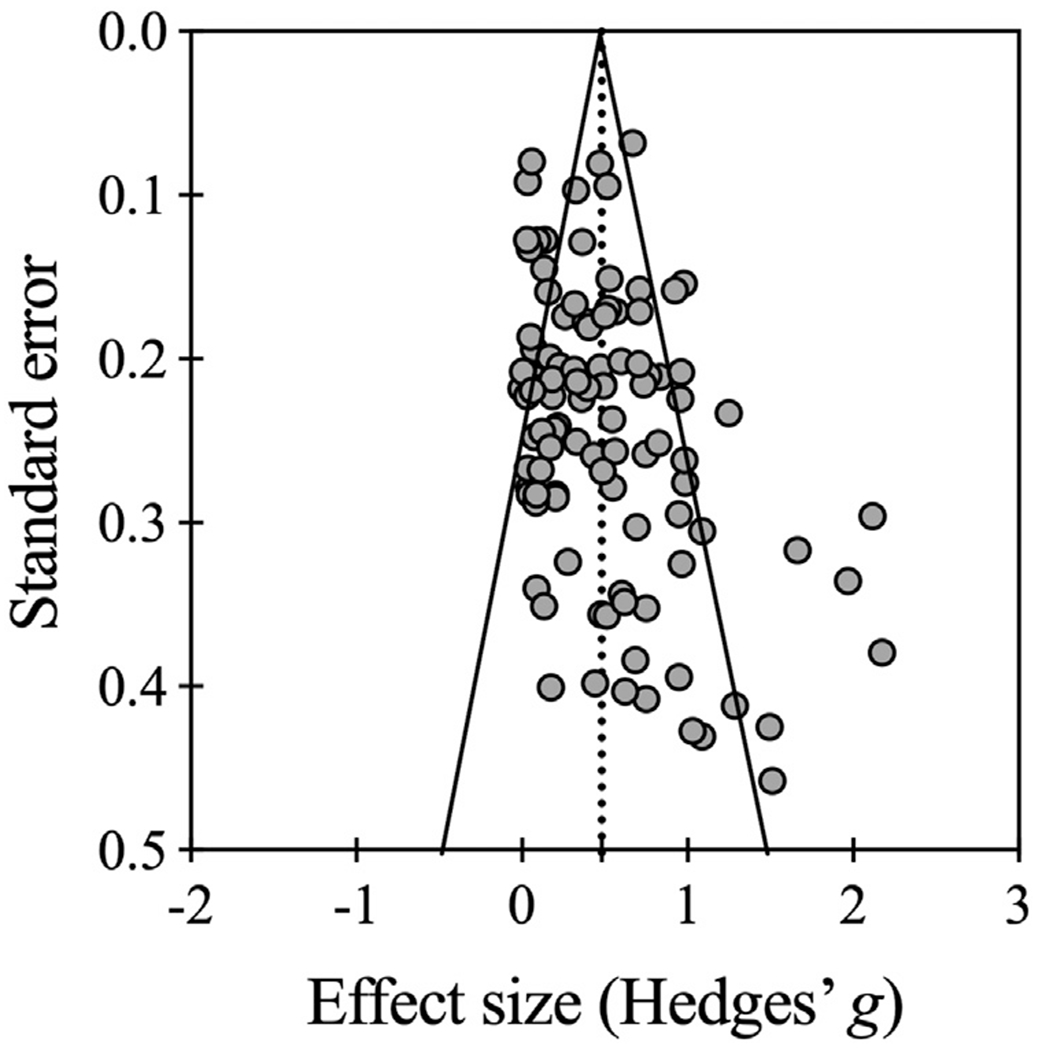
Funnel plot for publication bias assessment.

The Egger’s regression intercept suggested a significant risk of publication bias (2-tailed *P*=.003; 1-tailed *P*=.002), although the funnel plot showed an asymmetrical distribution (Fig. 8). Thus, a series of in-depth analyses further scrutinized the level of significance to understand the degree of publication bias. The 102 articles contained a total of 283 balance-related variables, with an average of 2.77 variables per article (range: 1-12). Of these 283 variables, 211 (74.6%) showed significant improvements post-AVG intervention; 72 (25.4%) did not improve significantly. None of the balance outcome metrics actually deteriorated. When these analyses were conducted within each study, 69 (67.6%) articles displayed significantly better outcomes; in 11 (10.8%) articles the outcomes showed no significant change; 22 (21.6%) reported mixed findings; and none of the study (0%) reported significantly worse outcomes.

## Discussion

### Scope and effect of this meta-analysis

This meta-analysis intended to assess the effect of AVGs on improving balance ability in a wide range of populations. Our synthesis was unique as our review included 129 articles with 6407 participants with differences in age and health conditions, intervention durations, and intervention settings, and gaming platforms. The most important finding was that AVGs provided a significant beneficial effect on balance ability, not only compared with passive control groups, but also compared with conventional therapy. While individuals without specific health conditions benefitted most, several other populations with neurologic conditions showed also significant improvements. The finding that the positive effects on postural balance can even surpass some conventional treatments may be of interest to health care providers to consider AVGs as a promising therapeutic option.

### Participant details

The included studies originated from 6 regions and 36 countries, with almost half of them conducted in Asia. This suggests that, although AVGs are pervasive and available for use globally, they are adopted more widely in Asia. This disproportionate usage may also simply be ascribed to the fact that Asia has about 60% of the world’s population (Van Bavel, 2013) with increasingly aging societies that contribute more seniors and elderly to the world’s population (Jarzebski, 2021).

The participants’ average age was 55.1 years, indicating that there were more articles with an older adult population. However, this may be an overly conservative number. For studies that only provide the age range without mean and standard deviation, we have only used the lower bound of the age range in our calculations. And yet, older adults have more balance problems: out of the 33 million adults in the US that reported balance problems, 26% were 65 years or older.^36^ However, even though balance ability decreases with age, children should also be represented as 3.3 million children in the US face balance issues.^37^ Although the average percentage of men participants was 40%, both sexes were represented. Of those with neurologic balance problems, 48.9% of children and 63.9% adult participants were women, suggesting the need for addressing specific balance problems in women as they age.^36^

The average number of the experimental group participants was 24 per study, with the range spanning 10-508 individuals. This small average number is likely a result of the costly nature of longitudinal balance studies (time, resources, personnel), especially if a large number of participants is recruited. In addition, some of the new technologies involved in these interventions pose significant monetary costs that increase with participant number. Another pertinent practical problem is that by the time researchers have obtained funding, the acquired consoles and games may no longer be relevant or supported by the developers.

### Benefits for different medical conditions

Our results indicated that multiple groups could reap benefits of AVGs on balance control, including those with a variety of neurologic impairments and those without. More than half of our studies involved people with 1 of 5 serious health conditions, with at least 3 studies per condition: stroke, Parkinson’s disease, multiple sclerosis, cerebral palsy, and diabetes. In addition, 21% of our studies tested other neurologic conditions such as fibromyalgia and unilateral peripheral vestibular deficit. Interestingly, the positive influence of AVGs on balance parameters cut across the different neurologic conditions. This reflects the all-encompassing nature of postural balance and the fact that balance problems are inherent to many neurologic problems. It is also not surprising that unimpaired individuals could benefit from AVG play. Around a quarter of our studies were conducted in healthy individuals and indicated improvements in balance ability. Public health officials may find these results of interest as AVGs can be implemented throughout society, ultimately to preemptively address the major issue of falling.

Our additional subgroup analyses provided more insights with clinical relevance. First, results revealed that AVG interventions were able to improve balance outcomes with similar or better efficiency than conventional training/therapy, regardless of the participants’ medical conditions. Such improvements in balance outcomes were independent of the different intervention environments. Even when the training was performed at home without direct supervision by a therapist, the results were similarly effective as in laboratory or clinical practice settings. While most AVG interventions for clinical populations were still conducted in a non-home-based environment (45 vs 7 studies), a main advantage of AVG interventions is the possibility to be used in patients’ homes, which holds promise for their wider application.

### Comment on intervention length

Given the significant improvements in balance ability, it is worth considering that the average intervention duration was 35.6 minutes per session for 3.1 sessions per week over 7.6 weeks. Our analyses also indicated that intervention length was an important factor to improve balance in healthy subjects and stroke patients, but the intervention length effect seemed to be inconclusive among Parkinson’s disease patients. Compared with other controlled therapeutic interventions, this seems to be a long period to elicit noticeable changes. This slow change is probably because balance is a sensorimotor ability that is reliant on all sensory modalities and coordinative abilities.

One unaddressed concern is the question to what degree improved balance persists beyond the training period. To answer this question, the intervention studies would have to include retention tests, with 1 or more weeks of no training after the interventions. Motor learning studies often include such tests to assess lasting adaptations in the central nervous system.^38^ As the current intervention studies did not address this point, this highly relevant question needs to be left for future work.

Nevertheless, our finding that about 2 months of training produced significant beneficial effects, sometimes even better than conventional therapy, is noteworthy. It is not unreasonable to expect that longer-term interventions with more intensive sessions may potentially amplify the balance improvements even further. Given the high motivational value and the continuous development of gaming technology, one may even consider the optimal scenario where these AVGs become a constant companion in everyday life.

### Tailoring of AVGs in collaboration between scientists, health care providers, and developers

The current popularity of video games is a fertile ground for developing more commercially available AVGs. However, commercial game development may also limit researchers in their ability to control the design of these interventions. Given the funds required to develop a game, clinicians and therapists may have to use pre-produced games for their interventions. However, development of specific game versions should proceed in communication with researchers, especially when clinical applications are intended. Ideally, game developers, health care providers, and scientists should jointly work on AVG designs to achieve maximally efficient, effective, and safe technology. Hospital databases can serve as sources for participant recruitment, thereby potentially engaging patients in tailoring their treatments. Meanwhile, as gaming and its industry are flourishing, scientists are accumulating a rich database for further work.

### Limitations

We included only articles that were written in English. Given that half of the included articles originated in Asia, the exclusion of non-English articles may have influenced our results. Extending the scope to include more languages should be a future goal. Further, our review included studies with relatively low GRADE scores, that is, were of relatively low scientific quality, and high-quality studies might have provided more reliable assessments of the AVG effects on balance ability. Hence, future researchers may conduct a meta-analysis of only high-quality controlled studies.

It is worth mentioning that, despite our efforts to objectively characterize the studies’ features and efficacy, our Egger’s test and the funnel plot indicated that publication bias could not be ruled out. The lower-left quarter of the funnel plot (negative effects with high standard error) is not populated, which is indicative of potential publication bias. Further examination suggested that almost 3 quarters of the 283 balance-related outcomes exhibited significant improvement and 67.6% of the 102 studies demonstrated significantly better outcomes. Only around 22% studies reported mixed findings, only around 11% studies did not elicit significant improvement, and none of the interventions led to deteriorated performance. Although this speaks to overall beneficial effects, it also suggests that non-significant and significantly worse outcomes may have either not been submitted for publication or been rejected in the review process. Yet, it is difficult or practically impossible to overcome this hurdle. We would encourage a balanced reporting of findings to achieve realistic insights into the effectiveness of balance training with AVGs.

Unfortunately, we were unable to differentiate between balance improvements in patients with different levels of severity of impairments. This limitation is set by the meta-analysis method, which uses individual articles as the unit of analysis. Most of the studies tended to group individuals with different severity of a certain neurologic condition into the same article. We hope future intervention studies will adopt a more differentiated criterion for participant recruitment and conduct specialized studies, for example, on only mild or moderate or severe cases. This would allow future synthesis studies to provide more precise estimation of the intervention efficacy as a function of the condition severity. Similarly, we hope future research on balance ability will refine precision and ease of measures and develop more economical test options with good reliability and validity for different scenarios. This would be a key factor in improving many studies’ scientific quality and replicability.

Last but not least, because many of the studies used more than 1 game, we were unable to distinguish individual game efficacy unless we had excluded those studies. However, this would have significantly limited the scope of our review.

## Conclusion and open questions

We have presented a systematic review and meta-analysis of 129 and 102 studies (respectively) published between January 1, 2009, and December 31, 2020, that used AVGs to enhance control of postural balance. We unequivocally found that AVGs provided a significant positive effect on balance performance. Despite this evidence, future researchers should seek to explore AVG interventions over a longer period and, critically, also evaluate the retention of the enhanced balance ability. If in many cases the regular use of AVGs cannot be expected, how long does the positive effect last? Other critical questions to be pursued are: Does the number of falls decrease? Is there a different effect in populations with degenerative diseases vs unimpaired individuals? Further, what potentially positive effects does an improved balance ability have on the quality of life? Collaboration between researchers, health care professionals, and game developers may maximize AVG development efficiency, produce AVGs for populations with different health issues, and enhance the quality of the AVG experience for all users. Nevertheless, the current positive AVG effects on postural balance hold promise for their more wide-spread use in physical therapy and rehabilitation.

## Supplementary Material

MMC1

MMC2

## Figures and Tables

**Table 1:** PRISMA 2020 Item Checklist

Section & Topic	Item	Checklist item	Location Where Item is Reported

TITLE			
Title	1	Identify the report as a systematic review.	1
ABSTRACT			
Abstract	2	See the PRISMA 2020 for Abstracts checklist	1
INTRODUCTION			
Rationale	3	Describe the rationale for the review in the context of existing knowledge.	4-7
Objectives	4	Provide an explicit statement of the objective(s) or question(s) the review addresses.	7
METHODS			
Eligibility criteria	5	Specify the inclusion and exclusion criteria for the review and how studies were grouped for the syntheses.	7-8
Information sources	6	Specify all databases, registers, websites, organizations, reference lists and other sources searched or consulted to identify studies. Specify the date when each source was last searched or consulted.	9-10
Search strategy	7	Present the full search strategies for all databases, registers and websites, including any filters and limits used.	9, Supplementary Material 1
Selection process	8	Specify the methods used to decide whether a study met the inclusion criteria of the review, including how many reviewers screened each record and each report retrieved, whether they worked independently, and if applicable, details of automation tools used in the process.	10-12
Data collection process	9	Specify the methods used to collect data from reports, including how many reviewers collected data from each report, whether they worked independently, any processes for obtaining or confirming data from study investigators, and if applicable, details of automation tools used in the process.	10-12
Data items	10a	List and define all outcomes for which data were sought. Specify whether all results that were compatible with each outcome domain in each study were sought (eg, for all measures, time points, analyses), and if not, the methods used to decide which results to collect.	10-12
	10b	List and define all other variables for which data were sought (eg, participant and intervention characteristics, funding sources). Describe any assumptions made about any missing or unclear information.	8-12
Study risk of bias assessment	11	Specify the methods used to assess risk of bias in the included studies, including details of the tool(s) used, how many reviewers assessed each study and whether they worked independently, and if applicable, details of automation tools used in the process.	11-12
Effect measures	12	Specify for each outcome the effect measure(s) (eg, risk ratio, mean difference) used in the synthesis or presentation of results.	12-13
Synthesis methods	13a	Describe the processes used to decide which studies were eligible for each synthesis (eg, tabulating the study intervention characteristics and comparing against the planned groups for each synthesis [item #5]).	10-13
	13b	Describe any methods required to prepare the data for presentation or synthesis, such as handling of missing summary statistics, or data conversions.	10-13
	13c	Describe any methods used to tabulate or visually display results of individual studies and syntheses.	10-13
	13d	Describe any methods used to synthesise results and provide a rationale for the choice(s). If meta-analysis was performed, describe the model(s), method(s) to identify the presence and extent of statistical heterogeneity, and software package(s) used.	10-13
	13e	Describe any methods used to explore possible causes of heterogeneity among study results (eg, subgroup analysis, metaregression).	12-13
	13f	Describe any sensitivity analyses conducted to assess robustness of the synthesised results.	10-13
Reporting bias assessment	14	Describe any methods used to assess risk of bias due to missing results in a synthesis (arising from reporting biases).	11-12
Certainty assessment	15	Describe any methods used to assess certainty (or confidence) in the body of evidence for an outcome.	10-13
RESULTS			
Study selection	16a	Describe the results of the search and selection process, from the number of records identified in the search to the number of studies included in the review, ideally using a flow diagram.	9-10, 13
	16b	Cite studies that might appear to meet the inclusion criteria, but which were excluded, and explain why they were excluded.	N/A
Study characteristics	17	Cite each included study and present its characteristics.	Supplementary Material 2
Risk of bias in studies	18	Present assessments of risk of bias for each included study.	15-16
Results of individual studies	19	For all outcomes, present, for each study: (a) summary statistics for each group (where appropriate) and (b) an effect estimate and its precision (eg, confidence/credible interval), ideally using structured tables or plots.	13-15, 16-18
Results of synthesis	20a	For each synthesis, briefly summarize the characteristics and risk of bias among contributing studies.	15-16
	20b	Present results of all statistical syntheses conducted. If meta-analysis was done, present for each the summary estimate and its precision (eg, confidence/credible interval) and measures of statistical heterogeneity. If comparing groups, describe the direction of the effect.	18-24
	20c	Present results of all investigations of possible causes of heterogeneity among study results.	18-24
	20d	Present results of all sensitivity analyses conducted to assess the robustness of the synthesized results.	10-13, Supplementary Material 3
Reporting biases	21	Present assessments of risk of bias due to missing results (arising from reporting biases) for each synthesis assessed.	23-24
Certainty of evidence	22	Present assessments of certainty (or confidence) in the body of evidence for each outcome assessed.	18-24
DISCUSSION			
Discussion	23a	Provide a general interpretation of the results in the context of other evidence.	24-28
	23b	Discuss any limitations of the evidence included in the review.	28-30
	23c	Discuss any limitations of the review processes used.	28-30
	23d	Discuss implications of the results for practice, policy, and future research.	30
FUNDING			
Registration and protocol	24a	Provide registration information for the review, including register name and registration number, or state that the review was not registered.	1
	24b	Indicate where the review protocol can be accessed, or state that a protocol was not prepared.	1
	24c	Describe and explain any amendments to information provided at registration or in the protocol.	N/A
Support	25	Describe sources of financial or non-financial support for the review, and the role of the funders or sponsors in the review.	Title page
Competing interests	26	Declare any competing interests of review authors.	Title page
Availability of data, code and other materials	27	Report which of the following are publicly available and where they can be found: template data collection forms; data extracted from included studies; data used for all analyses; analytical code; any other materials used in the review.	Title page
PRISMA 2020 for Abstract checklist SECTION & TOPIC	ITEM	CHECKLIST ITEM	REPORTED ON PAGE #
TITLE			
Title	1	Identify the report as a systematic review.	1
BACKGROUND			
Objectives	2	Provide an explicit statement of the main objective(s) or question(s) the review addresses.	1
METHODS			
Eligibility criteria	3	Specify the inclusion and exclusion criteria for the review.	1
Information sources	4	Specify the information sources (eg, databases, registers) used to identify studies and the date when each was last searched.	1
Risk of bias	5	Specify the methods used to assess risk of bias in the included studies.	1
Synthesis of results	6	Specify the methods used to present and synthesise results.	1
RESULTS			
Included studies	7	Give the total number of included studies and participants and summarize relevant characteristics of studies.	1
Synthesis of results	8	Present results for main outcomes, preferably indicating the number of included studies and participants for each. If meta-analysis was done, report the summary estimate and confidence/credible interval. If comparing groups, indicate the direction of the effect (ie, which group is favored).	1
DISCUSSION			
Limitations of evidence	9	Provide a brief summary of the limitations of the evidence included in the review (eg, study risk of bias, inconsistency and imprecision).	1
Interpretation	10	Provide a general interpretation of the results and important implications.	1
OTHER			
Funding	11	Specify the primary source of funding for the review.	Title page
Registration	12	Provide the register name and registration number.	1

Source: Page MJ, Moher D, Bossuyt P, et al. PRISMA 2020 explanation and elaboration: updated guidance and exemplars for reporting systematic reviews. doi:10.31222/osf.io/gwdhk.

See prisma-statement.org.

**Table 2:** Summary of balance outcome measure categories

	Categories	Definitions
1	Questionnaire/Clinical Scores	Experimenter/clinician/therapist measures a range of functional behaviors using an integer score that is compared with normative data; standardized questionnaires include selfreport or reports by caregivers
2	Functional Balance	Functional balance, including walk and turn, sit and stand, and so on, measured using simple quantitative metrics, for example, seconds, distance reached, number of repetitions
3	Functional Balance+	More complex functional balance involving head turns or dual cognitive tasks measured using simple quantitative metrics
4	Posturography	Fine-grained quantitative measures of static balance via CoP or CoM assessed by forceplates, usually with eyes open or eyes closed
5	Posturography+	Fine-grained quantitative measures of static balance via CoP or CoM during dynamic balance tasks guided by videos quantified by forceplates (CoP) typically provided by AVGs

*Abbreviation:* CoM, center of mass.

## List of abbreviations:

AVG: active video game
CI: confidence interval
CoP: center of pressure
GRADE: Grading of Recommendations Assessment, Development and Evaluation
PRISMA: Preferred Reporting Items for Systematic Reviews and Meta-analyses
PROSPERO: International Prospective Register of Systematic Reviews
SMD: standardized mean differences
TUG: timed Up and Go test
US: United States
