# Supplementary material for: A Systematic Review and Meta-analysis of the Effect of Active Video Games on Postural Balance
Source: Arch Phys Med Rehabil. Author manuscript; Available in PMC 2023 Apr 28. (PMC10142571; doi:10.1016/j.apmr.2023.01.002)
Supplement: MMC2 [file NIHMS1866529-supplement-MMC2.docx]

Balance Measures Table

| Study/Country | Study Design | Sample | | | | | Intervention | | | | Control group | Outcome |
| --- | --- | --- | --- | --- | --- | --- | --- | --- | --- | --- | --- | --- |
|  |  | E | C | Male % | Age | Condition | Game(s) | Platform | Session (min)/ Weekly Freq | Total Length (min)/ Week |  |  |
| Adcock, M., et al. 2020/ Switzerland* | Randomized pre-test post-test with control | *15* | *16* | 48 | *73.9±6.4* | NSC | Active @ Home | TV | 30/3 | 1440/16 | Nothing | ⭤ (SPPB) |
| AlSaif, A. A., et al. 2015/ Saudi Arabia | Randomized pre-test post-test with control | *20* | *20* |  | *6-10 years old* | CP |  | Wii | 20/7 | 1680/12 | Nothing | 🡑 (MABC-2 total)  🡑 (MABC-2 balance) |
| Anson, E., et al. 2018/US | Assessor blinded RCT | *20* | *20* | 28 | *75.8±5.9* | NSC | Visual feedback balance | Treadmill, TV | 16/3 | 360/4 | CT | 🡑 (BESTest)  ⭤ (BBS)  ⭤ (TUG) |
| Bang, Y. S., et al. 2016/South Korea | Randomized pre-test post-test with control | *20* | *20* |  | *62.7±6.3* | Stroke | Yoga, Muscular strength exercise,  Balancing exercise | Wii | 40/3 | 960/8 | Treadmill | 🡑 (Left/Right weight-bearing)  🡑 (A/P weight-bearing) |
| Barcala, L., et al. 2013/ Brazil | RCT | *10* | *10* | 45 | *64.4±13.5* | Stroke | Plata formas, Pesca Bajo cero, La cuerda floja | Wii | 30/2 | 900/5 | CT | 🡑 (Peak plantar pressure on parietic side)  🡑 (Peak plantar pressure on non-parietic side)  🡑 (ML oscillation with EO)  🡑 (ML oscillation with EC)  🡑 (A/P oscillations with EO)  🡑 (A/P oscillations with EC)  🡑 (Oscillations from COP with EO)  🡑 (Oscillations from COP with EC)  🡑 (BBS)  🡑 (TUG) |
| Barry, G., et al. 2016/UK | Randomized pre-test post-test with control | *24* | *23* | 43 | *33.0±12* | NSC | Reflex Ridge, River Rush, Boxing, Super Saver, Target Kick, Bump Bash | Xbox Kinect | 30/3 | 360/4 | Traditional Gym Based Exercise | 🡑 (A/P SD)  🡑 (A/P Range)  🡑 (ML SD)  🡑 (ML Range) |
| Cai, H., et al.  2019/China | Non-randomized controlled trial | *27* | *28* | 44 | *64.0 ±3.59* | Type II Diabetes | Kaimai-Style Qigong | Kinect | 30/3 | 1080/12 | Nothing | 🡑 (BBS) |
| Chao, Y.-Y., et al. 2014/US | Quasi-experimental  pre-post | *15* | *15* | 25 | *85.2 ±6.47* | Assisted living | Basic Run , Table Tilt, Lunge, Chair, Penguin Slide, Deep Breathing | Wii | 30/2 | 480/4 | Nothing | 🡑 (BBS)  🡑 (TUG) |
| Chen, C. L., et al. 2012/ Taiwan | RCT | *13* | *15* | 68 | *8.59±2.15* | CP | SimCycle | Eloton | 20/3 | 1440/12 | CT | ⭤ (BOTMP balance) |
| Cho, G. H., et al. 2014/South Korea | Randomized pre-test post-test with control | *17* | *15* |  | *72.4±1.15* | NSC | Ski Slalom, Table Tilt, Balance Bubble | Wii | 30/3 | 720/8 | Nothing | 🡑 (COP excursion area EO)  🡑 (COP excursion area EC) |
| Cho, H., et al.  2014/South Korea | Randomized pre-test post-test with control | *23* | *23* | 61 | *59.3±8.2* | End stage renal failure | Hula Hoop, Dance Step, Rhythm Parade, Twist Upper Body, One Arm Pull Back, Chair Position, Half Moon Position, Flying Game, Twist, Rhythm Kung Fu, Knee Flexion and Pull, Balance Walk, Mii Triathlon, Balance Bead, Juggling, Leg Wide Open Side Flexion, Lifting the Side Limbs, Knee Hugging Position | Wii | 40/3 | 960/8 | Nothing | 🡑 (Balance) |
| Cho, K. H., et al. 2012/South Korea | Randomized pre-test post-test with control | *11* | *11* | 64 | *64.2±7.61* | Stroke | Balance Bubble, Ski Slalom, Ski Jump, Penguin Slide, Soccer Heading, Table Tilt | Wii | 30/3 | 2340/6 | CT | ⭤ (PSV-A/P EO)  ⭤ (PSV-A/P EC)  ⭤ (PSV-ML EO)  ⭤ (PSV-ML EC)  🡑 (BBS)  🡑 (TUG) |
| Choi, D., et al.  2018/South Korea | RCT | *14* | *14* | 61 | *50.3±18.4* | Stroke | Ski slalom, Table Tilt, Tightrope Walking, Soccer Heading, Penguin Slide, Snowboard Slalom | Wii | 30/3 | 2160/6 | CT | 🡑 (BBS)  🡑 (TUG) |
| Choi, W., et al. 2019/South Korea | RCT | *30* | *30* | 15 | *76.3 ±4.17* | NSC | Virtual Kayak Paddling |  | 40/2 | 720/6 | CT | 🡑 (EO ML sway)  🡑 (EO A/P sway)  🡑 (EC ML sway)  🡑 (EO A/P sway)  🡑 (Right OLS)  🡑 (Left OLS)  🡑 (TUG)  🡑 (BBS)  🡑 (FSST) |
| Chow, D. H. K., et al. 2015/Hong Kong | Pilot RT | *20* | *20* | 35 | *69.0±4.2* | NSC | Tiger Woods PGA Tour 13 | Xbox 360 Kinect | 45/7 | 630/2 | Sedentary Games | ⭤ (TUG)  🡑 (SLT) |
| Cikajlo, I., et al. 2020/ Slovenia | Pilot RT | *10* | *10* | 75 | *51.1 ±11.7* | Stroke | Animal Hurdler, Fruit Catcher, Horse Runner | Wii | 15/5 | 75/1 | CT | 🡑 (TUG)  🡑 (FSST)  ⭤ (CTSIB EO)  ⭤ (CTSIB EC  ⭤ (STOLL EO)  ⭤ (STOLL EC)  ⭤ (STORL EO)  🡑 (STORL EC)  ⭤ (sROM EO  🡑 (sROM EC) |
| Cimino, V., et al. 2019/Italy | Randomized pre-test post-test with control | *19* | *27* | 41 | *51.2±11.2* | MS | Penguin Slide, Ski Slalom, Perfect 10, Heading, Table Tilt, Tightrope Tension, Balance Bubble, Snowboard Slalom, Table Tilt+, Skateboard Arena, Balance Bubble+ | Wii | 60/5 | 1200/4 | CT | 🡑 (BBS)  🡑 (BI) |
| Da Fonseca, E. P., et al.  2017/Brazil* | RCT | *13* | *14* | 37 | *52.4±8.9* | Hemiparesis | Tennis, Hula Hoop | Wii | 45/2 | 1200/10 | CT | ⭤ (DGI)  🡑 (Occurrence of Falls) |
| Ditchburn, J.-L., et al. 2019/UK | Randomized pre-test post-test with control | *27* | *27* | 78 | *71.0±5.0* | Chronic Musculo-skeletal Pain | Volleyball, Sharkbait, Formula Racing, Snowboard, Birds and Balls | Interactive Rehab. and Exercise System | 40/2 | 480/6 | CT | 🡑 (PS-A/P EO SD)  🡑 (PS-A/P EC range)  🡑 (PS-ML EC SD)  ⭤ (PS-ML EO range)  ⭤ (PSV-COP EO)  ⭤ (PS-A/P EO SD)  ⭤ (PS-A/P EC range)  ⭤ (PS-ML EO SD)  ⭤ (PS-ML EC range)  ⭤ (PSV-COP EC) |
| Duque, G., et al. 2013/ Australia* | Randomized pre-test post-test with control | *30* | *40* | 39 | *76.8±8.86* | Falls | Linear Bars, Saccadic, Tren Optokinetic, VOR Suppression, Linear Train, Optokinetic Tunnel, Maze, Breakfast, Surfing | Balance Rehab. Unit | 30/2 | 360/6 | CT | 🡑 (LOS)  🡑 (EO on Hard Surface)  🡑 (EC on Hard Surface)  🡑 (EC on Foam)  🡑 (Vertical VVC) |
| Eftekharsadat, B., et al.2015/ Iran | RCT | *15* | *15* | 27 | *35.2±8.2* | MS | Postural Stability Training Program (PST) | Biodex Balance System SD | 20/2 | 480/12 | Nothing | 🡑 (TUG)  ⭤ (BBS)  🡑 (OSI) |
| Fakhro, M. A., et al. 2019/ Lebanon* | RCT | *33* | *31* |  | *72.2±5.2* | Institutionalized | Soccer Heading, Table Tilt | Wii Fit Balance Board | 40/3 | 960/8 | Nothing | 🡑 (TUG)  🡑 (COP) |
| Feng, H., et al. 2019/China | RCT | *14* | *14* | 57 | *67.2±4.72* | PD | Hand and feet touch the ball, Hard boating, Take the maze, Cool-down | VR | 45/5 | 2700/12 | CT | 🡑 (BBS)  🡑 (TUG)  🡑 (FGA) |
| Ferguson, G. D., et al. 2013/South Africa | Pragmatic Quasi-Experimental Study | *19* | *27* | 52 | *8.22±1.34* | DCD | Cycling, Soccer, Skateboarding, Skiing | Wii | 30/3 | 540/6 | CT | 🡑 (MABC-2 total)  🡑 (MABC-2 balance) |
| Fitzgerald, D., et al. 2010/ Ireland | RCT | *14* | *14* | 45 | *26.2±2.65* | NSC | Neverball | Wobble Board, Computer | 15/3 | 180/4 | CT | ⭤ (Anterior SEBT)  🡑 (Posteromedial SEBT)  🡑 (Posterolateral  SEBT)  ⭤ (DPSI) |
| Franco, J. R., et al. 2012/US | Randomized pre-test post-test with control | *11* | *21* | 22 | *78.3±6* | NSC | Soccer Heading, Ski Jumping, Ski Slalom, Table Tilt, Tightrope, Balance Bubble | Wii | 13/2 | 163/3 | Nothing and CT | ⭤ (BBS)  ⭤ (TGBA) |
| Fritz, S. L., et al. 2015/US | RCT | *16* | *14* |  | *66.2±9.67* | Stroke | Wii Sports, Wii Fit, EyeToy Play 2, Kinetic | Computer, Wii | 50/4 | 1000/5 | Nothing | ⭤ (BBS)  ⭤ (DGI) |
| Gandolfi, M., et al. 2017/ Italy | RCT | *38* | *38* | 67 | *68.6±8.3* | PD | Table Tilt, Penguin Slide, Balance Bubble, Ski Slalom, Skateboarding, Perfect 10, Tilt City, Snowball Fight, Rhythm Parade, Bird’s- Eye Bulls-Eye | Wii | 50/3 | 1050/7 | CT | 🡑 (BBS) |
| Gatica-Rojas, V., et al.  2017/Chile* | RCT | *16* | *16* | 59 | *10.7±3.35* | CP | Snowboard, Penguin Slide, Super Hula Hoop | Wii | 30/3 | 540/6 | CT | 🡑 (CoP sway EO)  🡑 (CoP sway EC) |
| Gioftsidou, A., et al. 2013/Greece | Randomized pre-test post-test with control | *20* | *20* | 55 | *20.4±0.68* | NSC | Ski Slalom, Table Tilt, Balance Bubble, Penguin Slide, Skateboard Arena, Balance Headings, Headings, Snowboard Slalom, Balance Bubble +, Table Tilt + | Wii | 24/2 | 384/8 | CT | 🡑 (Biodex SI Right Leg)  🡑 (Biodex SI Left Leg)  🡑 (Biodex A/P Index Right Leg)  🡑 (Biodex A/P Index Left Leg)  🡑 (Biodex MLI Right Leg)  🡑 (Biodex MLI Left Leg)  🡑 (Board 1a Right Leg)  🡑 (Board 1a Left Leg)  🡑 (Board 1b Right Leg)  🡑 (Board 1b Left Leg)  🡑 (Board 2 Right Leg)  🡑 (Board 2 Left Leg) |
| Gutierrez, R. O., et al. 2013/Spain | Randomized pre-test post-test with control | *25* | *25* | 43 | *41.2±7.76* | MS | Kinect Sports, Joy Ride, Adventures | Xbox 360 | 20/4 | 800/10 | CT | 🡑 (Compuesto-SOT)  🡑 (Vestibular Ratio)  🡑 (BBS)  🡑 (TT) |
| Hamari, L., et al. 2019/ Finland* | RCT | *17* | *19* | 72 | *7.8* | Cancer | Wii Fit | Wii | 30/7 | 1680/8 | CT | ⭤ (MABC-2) |
| Henrique, P.P.B., et al. 2019/Brazil | RCT | *16* | *15* | 45 | *76.2±10.2* | Stroke | Motion Rehab AVE 3D | Computer, Kinect | 30/2 | 720/12 | CT | 🡑 (BBS) |
| Huang, H.-C., et al. 2017/ Taiwan* | RCT | *55* | *62* | 43 | *22.7±2.1* | NSC | Your Shape: Fitness Evolved | Xbox 360 | 30/3 | 1080/12 | Nothing | ⭤ (Balance) |
| Hung, E. S.-W., et al. 2019/Taiwan | Randomized crossover open-label | *12* | *12* | 33 | *68.8±1.66* | Diabetes | Stepping, hamsters, drumming | XavixPORT | 30/3 | 540/6 | Nothing | 🡑 (BBS)  ⭤ (TUG) |
| Hung, J.-W., et al. 2014/ Taiwan | RCT | *15* | *15* | 64 | *54.3±9.99* | Stroke | Table Tilt, Ski Slalom, Soccer Heading, Balance Bubble, Penguin Slide, Basic Step, Warrior | Wii | 30/2 | 720/12 | CT | 🡑 (TUG) |
| Hung, J.-W., et al. 2016/ Taiwan* | Randomized pre-test post-test with control | *14* | *13* | 70 | Over 18 years old | Stroke | Catch, Skyball, Tag, Gotcha, Speedball, Immobilizer, Target, Freeze | Tetrax Biofeedback Exercise System | 100/3 | 2160/6 | Nothing | 🡑 (TUG) |
| Ibrahim, M. S., et al. 2016/ Egypt | Randomized pre-test post-test with control | *15* | *15* | 47 | 41.8*±6.85* | NSC | Tight-rope walks, Balance Bubble, Soccer Headers, Penguin Slide, Table Tilt | Wii | 15/3 | 180/4 | CT | 🡑 (OLB) |
| Jorgensen, M. G., et al. 2013/ Denmark | RCT | *28* | *30* | 31 | *74.8±5.9* | NSC | Table tilt, Slalom Ski, Perfect 10, Tight Rope Tension, Penguin Slide | Wii | 35/2 | 1900/10 | Nothing | 🡑 (TUG) |
| Ju, Y.-J., et al. 2018/Taiwan | Randomized pre-test post-test with control | *12* | *24* | 50 | *7.03±1.42* | DCD | iBalance | Wii Fit Balance Board | 45/3 | 540/4 | Nothing | 🡑 (MABC-2)  🡑 (SLS)  🡑 (COP trajectory of SLS) |
| Jung, J., et al.  2012/South Korea | Randomized pre-test post-test with control | *11* | *10* | 62 | *62.0±6.85* | Stroke |  | VR treadmill | 30/5 | 450/3 | Treadmill without VR | 🡑 (TUG) |
| Kalron, A., et al. 2016/Israel | RCT | *16* | *16* | 37 | *45.6±10.1* | MS |  | Computer Assisted Rehab. Environment  System | 30/2 | 360/6 | CT | ⭤ (BBT)  🡑 (FSST) |
| Kannan, L., et al. 2019/US | RCT | *12* | *12* | 54 | *59.3±6.3* | Stroke | Bubble Balance, Table Tilt, Tight-Rope Walking, Soccer Head | Wii | 90/3.33 | 1798.2/6 | CT | 🡑 (BBS)  🡑 (TUG) |
| Karahan, A. Y., et al. 2015/Turkey | RCT | *54* | *46* | 57 | *71.4±5.4* | NSC | Kinect Adventures, Kinect Sports, Kinect Sports Season two | Xbox 360 Kinect | 30/5 | 900/6 | CT | 🡑 (BBS)  🡑 (TUG) |
| Karasu, A.U.,  et al.  2018/ Turkey | Randomized pre-test post-test with control | *12* | *11* | 43 | *63.1±12* | Stroke | Heading, Ski Slalom, Table Tilt, Tightrope Tension, Balance Bubble, Penguin Slide | Wii | 20/5 | 400/4 | CT | 🡑 (BBS)  🡑 (FRT)  🡑 (TUG)  🡑 (SBI) |
| Karssemeijer, E. G. A., et al. 2019/ Netherlands* | RCT | *38* | *77* | 62 | *79.2±6.9* | Dementia | Bike Labyrinth | Bicycle | 30/3 | 1080/12 | Nothing and CT | ⭤ (TUG) |
| Katajapuu, N., et al. 2017/ Singapore* | Randomized pre-test post-test with control | *10* | *20* | 30 | *71.3±6.62* | NSC | Skiing, Hiking, Pikkuli, China Town Race, RecReha | Microsoft  Kinect | 50/2 | 600/6 | Nothing and CT | ⭤ (BBS) |
| Khurana, M., et al. 2017/ India | Randomized pre-test post-test with control | *15* | *15* | 93 | *29.6±7.4* | Paraplegia | Birds and Balls, Soccer, Snowboard | Playstation 2 | 45/5 | 900/4 | CT | 🡑 (T-shirt Test) |
| Khushnood, K., et al. 2019/Pakistan | RCT | *33* | *33* | 74 | 45-70 years old | Diabetes | Wii Fit training | Wii | 30/2 | 480/8 | CT | 🡑 (BBS)  🡑 (TUG) |
| Kim, J., et al.  2013/South Korea | Controlled Cohort | *18* | *18* | 16 | *67.3±3.81* | NSC | Your Shape Fitness Evolved | Xbox 360 | 60/3 | 1440/8 | Nothing | 🡑 (BST with EC)  🡑 (BST with EO)  🡑 (CST with EC)  🡑 (CST with EO) |
| Kim, J. H., et al. 2009/South Korea | RCT | *12* | *12* | 58 | *52.0±8.4* | Stroke | Stepping up/down, Sharkbait, Snowboard | IREX VR System | 30/4 | 480/4 | Nothing | 🡑 (BBS) |
| Kliem, A., et al. 2010/ Germany | Randomized pre-test post-test with control | *11* | *11* | 23 | *47.6±13.1* | NSC | Ski Slalom, Balance Bubble | Wii | 10/3 | 99/3 | CT | 🡑 (SEBT)  🡑 (Dynamic Balance) |
| Kramer, A., et al. 2014/ Germany | Matched Controlled Trial | *21* | *40* | 28 | *47.0±9* | MS | Tennis, Table Tennis, Boxing, Archery, Sword Fight, Ski Slalom, Balance Bubble, Penguin Picnic, Soccer Heading, Tilt City, Perfect Ten | Wii | 30/3 | 270/3 | CT | ⭤ (Romberg Stance EO)  🡑 (Romberg Stance EC) |
| Kwok, B. C., et al. 2016/ Singapore | RCT | *40* | *40* | 15 | *70.2±7.1* | NSC | Nintendo Wii Active | Wii | 20/1 | 240/12 | CT | 🡑 (TUG) |
| Lai, C.- H., et al. 2013/ Taiwan | Randomized crossover | *15* | *15* | 43 | *72.1±4.8* | NSC |  | Xavix Measured Step System | 30/3 | 540/6 | Nothing | 🡑 (BBS)  🡑 (TUG)  ⭤ (UST) |
| Laver, K., et al. 2012/ Australia | RCT | *22* | *22* | 21 | *84.9±4.5* | NSC | Wii Fit | Wii | 25/5 | 250/2 | CT | 🡑 (TUG) |
| Lee, C.-H., et al. 2014/South Korea | RCT | *10* | *11* | 67 | *51.1±12* | Stroke |  | AR | 30/3 | 360/4 | Nothing | 🡑 (TUG)  🡑 (BBS) |
| Lee, H.-C., et al. 2017/ Taiwan | RCT | *26* | *24* | 72 | *57.7±9.3* | Stroke | Kinect Sports, Kinect Adventures, Your Shape Fitness | Microsoft Kinect | 45/2 | 1080/6 | CT | 🡑 (BBS)  🡑 (TUG) |
| Lee, H. Y., et al. 2015/South Korea | Randomized pre-test post-test with control | *12* | *12* | 67 | *47.5±12.6* | Stroke | Sitting Posture, Knee Bend and Other Leg Knee Extend, Walking a Tightrope, Penguin Teeter-Totter Seesaw, Balance Skiing, Rolling Marble Board, Balance Mii | Wii | 30/3 | 540/6 | CT | 🡑 (COP Path Length EO)  🡑 (COP Velocity EO)  🡑 (COP Pressure Length EC)  🡑 (COP Velocity EC) |
| Lee, I. W., et al. 2015/South Korea | Randomized pre-test post-test with control | *10* | *10* | 55 | *55±10.5* | Stroke | City Walking, Hot Air Balloon, Bubble | BioRescue | 45/3 | 810/6 | CT | 🡑 (BBS)  🡑 (TUG) |
| Lee, N. Y., et al. 2015/South Korea | Randomized pre-test post-test with control | *10* | *10* | 50 | *69.3±2.9* | PD | K-Pop Dance Festival | Wii | 30/5 | 900/6 | Nothing | 🡑 (BBS) |
| Lee, S., et al.  2013/South Korea | Randomized pre-test post-test with control | *27* | *28* | 29 | *74±5* | Diabetes | Wishi Washi: Window Washing, Keep Ups: Heading Game, Bowling, Bubble Pop, Boot Camp, Kung Foo | Playstation 2 | 50/2 | 1000/10 | Nothing | 🡑 (OLS)  🡑 (BBS)  🡑 (TUG) |
| Lee, Y., et al.  2017/South Korea | Randomized pre-test post-test with control | *22* | *22* | 43 | *75.9±4.73* | NSC | Step Dance, Ski Jump, Jogging, Swordplay, Hula-Hoop, Tennis | Wii | 60/2 | 720/6 | Nothing | 🡑 (BBS)  🡑 (TUG) |
| Leutwyler, H., et al. 2018/US | RCT | *13* | *15* | 86 | *38.0±9.5* | Schizophrenia | Bowling, Baseball, Skiing, Kinect Dance Central 2, Kinect Adventures, Kinect Your Shape Fitness Evolved | Xbox 360 | 30/1 | 180/6 | Sedentary Games | ⭤ (SPPB) |
| Liao, Y.-Y., et al. 2019/ Taiwan | RCT | *27* | *25* | 31 | *81.8±7* | Prefrail and frail elderly |  | Kinect | 60/3 | 2160/12 | CT | 🡑 (TUG) |
| Liao, Y.-Y., et al. 2019/ Taiwan | RCT | *18* | *16* | 32 | *74.4±6.0* | MCI |  | Kinect | 60/3 | 2160/12 | CT | 🡑 (FGA) |
| Liao, Y.-Y., et al. 2015/ Taiwan | RCT | *12* | *24* | 47 | *65.7±7.4* | PD | Soccer Heading, Marble Balance, Ski Slalom, Balance Bubble | Wii | 45/2 | 540/6 | Nothing and CT | 🡑 (TUG) |
| Lin, Y. T., et al. 2020/ Taiwan | RCT | *40* | *40* | 49 | *57.0±16.4* | Knee Osteoarthritis | Whack-a-Mole, Archery | Hot Plus System | 20/3 | 240/4 | CT | 🡑 (Balance) |
| Lloréns, R., et al. 2015/Spain | RCT | *10* | *10* | 45 | *56.7±11.6* | Stroke | VR-Stepping Exercise | OptiTrack | 30/5 | 600/4 | CT | 🡑 (BBS)  ⭤ (TT- Balance) |
| Martín-Martínez, J. P., et al. 2019/ Spain | RCT | *28* | *27* | 0 | *53.7±9.9* | Fibromyalgia | VirtualEX-FM |  | 60/2 | 3118/25.98 | Nothing | 🡑 (TUG) |
| McEwen, D., et al. 2014/ Canada | RCT | *30* | *29* | 54 | *64.1±15* | Stroke | Soccer Goaltending, Snowboarding | Interactive Rehab. Exercise | 20/3 | 180/3 | Sedentary Games | 🡑 (TUG) |
| Mombarg, R., et al. 2013/ Netherlands | Randomized pre-test post-test with control | *15* | *14* | 79 | *9.6±1.5* | Children with poor motor performance | Ski-jump, Segway Circuit, Obstacle Course, Skateboarding | Wii | 30/3 | 540/6 | Nothing | ⭤ (Static Balance MABC-2)  🡑(Walking on a Line MABC-2) |
| Montero-Alía, P., et al. 2019/ Spain | Quasi-randomized, open-label controlled clinical trial | *508* | *469* | 41 | *70 years or older* | NSC | Balance Bubble, Soccer Heading, Ski Jump, Table Tilt, Ski Slalom, Penguin Slide, Snowboard Slalom, Tightrope Walk | Wii Fit | 30/2 | 779.4/12.99 | Nothing | ⭤ (TT) |
| Morat, M., et al. 2019/ Germany | RCT | *17* | *34* | 38 | *69.4±5.6* | NSC | Targets, Divided, Simon, Flexi, Snake, Tetris, Habitats, Birds, Hexagon | Dividat Senso | 12/3 | 288/8 | Nothing | 🡑 (Y-balance- ANT)  🡑 (Y-balance-PM)  🡑 (Y-balance-PL)  🡑 (TUG-NT)  🡑 (TUG-DTm) |
| Morone, G., et al. 2016/Italy | RCT | *19* | *19* | 0 | *68.9±3.96* | Women with presence of bone loss | Balance Bubble, Ski Slalom, Table Tilt | Wii Fit | 60/2 | 960/8 | CT | 🡑 (BBS) |
| Morone, G., et al. 2014/Italy* | RCT | *25* | *25* |  | *60.2±10.0* | Stroke | Hula Hoop, Bubble Blower, Sky Slalom | Wii Fit | 20/3 | 240/4 | CT | 🡑 (BBS) |
| Morrison, S., et al. 2018/  US* | Randomized pre-test post-test with control | *31* | *34* | 58 | *67.3±5.32* | Type II Diabetes | Aerobics, Yoga, Balance, Strength Training | Wii | 40/3 | 1440/12 | CT | 🡑 (Falls Risk) |
| Mugueta-Aguinaga, L., et al. 2017/ Spain* | RCT | *20* | *19* | 41 | *84.3±7.7* | Frailty | FRED | Microsoft Kinect | 20/3 | 180/3 | Nothing | 🡑 (SPPB) |
| Nicholson, V. P., et al. 2015/  US* | Randomized pre-test post-test with control | *19* | *22* | 34 | *74.5±5.4* | NSC | Soccer Heading, Penguin Slide, Ski Slalom, Ski Jump, Table Tilt. Snowball Fight, Perfect 10, Tightrope Walking | Wii | 30/3 | 540/6 | Nothing | 🡑 (TUG)  🡑 (SLS left)  🡑 (SLS right) |
| Nilsagård, Y. E., et al. 2013/ Sweden | RCT | *42* | *42* | 24 | *49.7±11.3* | MS | Penguin Slide, Ski Slalom, Perfect 10, Heading, Table Tilt, Tightrope Tension, Balance Bubble, Table Tilt +, Balance Bubble +, Snowboard Slalom, Skateboard Arena | Wii | 30/2 | 360/6 | Nothing | ⭤ (TUG) |
| Ordnung, M., et al. 2017/ Germany* | Randomized pre-test post-test with control | *15* | *15* | 48 | *69.2±5.47* | NSC | Summer Stars 2012 | Xbox 360, Microsoft Kinect | 60/2 | 720/6 | Nothing | ⭤ (COP AP EO)  ⭤ (COP AP EC)  ⭤ (COP ML EO)  ⭤ (COP ML EC) |
| Padala, K. P., et al. 2017/ US* | Randomized controlled parallel-group | *15* | *15* | 63 | *73.0±6.2* | AD | Yoga, Strength Training, Aerobics, Balance Games, Training Plus | Wii | 30/5 | 1200/8 | CT | 🡑(BBS) |
| Padala, K. P., et al. 2012/US | Randomized pre-test post-test with control | *11* | *11* | 27 | *80.5±7.5* | AD | Soccer Heading, Ski Slalom, Ski Jump, Table Tilt, Balance Bubble, Penguin Slide | Wii | 30/5 | 1200/8 | CT | 🡑 (BBS)  🡑 (TT)  ⭤ (TUG) |
| Park, E. C., et al. 2015/South Korea | Randomized pre-test post-test with control | *12* | *12* | 79 | *65.9±8* | NSC | Soccer Heading, Snowboard Slalom, Table Tilt | Wii | 30/3 | 720/8 | Ball game exercise | 🡑 (Sway Length)  🡑 (TUG) |
| Park, J., et al. 2016/South Korea | Randomized pre-test post-test with control | *36* | *36* | 6 | *73.5±2.93* | Community-Dwelling Elderly | 3-D Virtual Reality Kayak | Projector | 20/2 | 240/6 | CT | 🡑 (X Sway EO Standing)  🡑 (Y Sway EO Standing)  🡑 (VM EO Standing)  🡑 (X Sway EC Standing)  🡑 (Y Sway EC Standing)  🡑 (VM EC Standing)  🡑 (X Sway EO Sitting)  🡑 (Y Sway EO Sitting)  🡑 (VM EO Sitting)  🡑 (X Sway EC Sitting)  🡑 (Y Sway EC Sitting)  🡑 (VM EC Sitting) |
| Pedreira da Fonseca, E., et al. 2017/ Brazil* | RCT | *14* | *13* | 30 | *52.4±8.9* | Stroke | Tennis, Hula Hoop | Wii | 45/2 | 1558.8/12.99 | CT | 🡑 (DGI) |
| Pompeu, J. E., et al. 2012/ Brazil | RCT | *16* | *16* | 53 | *67.4±8.1* | PD | Wii Fit | Wii | 30/2 | 840/7 | CT | 🡑 (BBS)  🡑 (UST EO)  ⭤ (UST EO, Dual Task)  🡑 (UST EC) |
| Portela, F. R., et al. 2011/ Portugal* | Randomized pre-test post-test with control | *23* | *42* | 38 | *79* | NSC | Wii Sports | Wii | 50/- | 1000/- | Gymnastics | ⭤ (BI)  ⭤ (BBS) |
| Prosperini, L., et al. 2013/ Italy | Randomized 2-Period Crossover Pilot | *18* | *18* | 31 | *36.2±8.7* | MS | Zazen, Table Tilt, Ski Slalom, Penguin Slide, Tightrope Walk, Soccer Heading, Balance Bubble | Wii | 30/5 | 1800/12 | Nothing | 🡑 (FSST) |
| Ribas, C. G., et al. 2017/ Brazil | RCT | *10* | *10* | 60 | *61.0±9.06* | PD | Table Tilt, Tilt City, Penguin Slide, Soccer Heading, Basic Run, Obstacle Course, Basic Step | Wii | 30/2 | 720/12 | CT | 🡑 (BBS) |
| Rica, R. L., et al. 2020/ Brazil* | Randomized pre-test post-test with control | *17* | *33* | 0 | *Over 60* | Institutionalized older adults | Kinect Sports Ultimate Collection, Your Shape Fitness Evolved, Dance Central, Nike + Kinect Training | Xbox 360 | 60/3 | 2219.4/12.33 | Nothing | 🡑 (Sit-and-stand test)  🡑 (Static Balance Test) |
| Rosiak, O., et al. 2018/ Poland* | Non-randomized Controlled Group | *25* | *25* | 48 | *46.5±10.6* | Unilateral Peripheral Vestibular Deficit | Meteorites | Hybrid VR Unit | 30/7 | 279.3/1.33 | CT | 🡑 (Quiet Stance EO COP Length)  ⭤ (Quiet Stance EO COP Surface)  🡑 (Quiet Stance EC COP Length)  🡑 (Quiet Stance EC COP Surface) |
| Rutkowski, S., et al. 2020/ Poland* | RCT | *38* | *68* | 43 | *61.3±3.6* | COPD | 20,000 Leaks, Curvy Creek, Rally Ball, Reflex Ridge | Xbox 360 Kinect | 20/5 | 500/2 | CT | ⭤ (TUG) |
| Şahin, S., et al. 2020/ Turkey | Single-blind RCT | *30* | *30* | 62 | *10.4±3.43* | Unilateral Spastic CP | Air Challenge, Boxing Trainer, Wall Breaker, Jet Run, Super Kick |  | 45/2 | 720/8 | CT | 🡑 (BOTMP total)  🡑 (BOTMP balance) |
| Salem, Y., et al. 2012/US | RCT | *20* | *20* | 55 | *4.05±0.48* | DD | Wii Sport, Wii Fit | Wii | 30/2 | 600/10 | CT | ⭤ (TUG)  🡑 (SLS) |
| Santos, P., et al. 2019/ Brazil | RCT | *13* | *28* | 76 | *63.2±8.55* | PD | Wii Sport, Wii Fit | Wii | 50/2 | 640/8 | CT | ⭤ (BBS)  ⭤ (DGI)  ⭤ (TUG) |
| Sato, K., et al. 2015/Japan* | RCT | *29* | *28* | 20 | *69.3±5.41* | NSC | Apple Game, Tightrope Standing, Balloon Popping, One-Leg Standing | Microsoft Kinect | 60/3 | 1440/8 | Nothing | 🡑 (BBS) |
| Schoene, D., et al. 2013/ Australia | RCT | *15* | *17* |  | *78.0±4.5* | NSC | Stepmania | Computer | 20/3 | 480/8 | Nothing | 🡑 (CSRT RT)  🡑 (CSRT MT)  🡑 (CSRT resp)  🡑 (TUG) |
| Sheehan, D. P., et al. 2013/ Canada | Pre-test post-test with non-equivalent control | *21* | *40* | 54 | *Fourth grade students* | NSC | iDance, XR Board/ Lightspace, Wii Fit Plus | Wii | 34/5 | 1020/6 | CT | 🡑 (PSC) |
| Shen, X., et al. 2015/ China* | RCT | *26* | *25* | 56 | *64.3±8.25* | PD | Computerized Dancing System, Smart-EquiTest Balance Master | Computer | 60/5 | 3900/13 | CT | 🡑 (SLS) |
| Shih, M.-C., et al. 2016/ Taiwan | Single-blinded RCT | *10* | *10* | 80 | *68.2±8.55* | PD | Reaching Task 1, Reaching Task 2, Obstacle Avoidance, Marching | Kinect | 30/2 | 800/8 | CT | 🡑 (LOS RT)  ⭤ (LOS MV)  🡑 (LOS Endpoint Excursion)  🡑 (LOS Directional Control)  ⭤ (OLS Less Affected EO)  ⭤ (OLS More Affected EO)  🡑 (OLS Less Affected EC)  ⭤ (OLS More Affected EC)  🡑 (BBS)  🡑 (TUG) |
| Silva, V., et al. 2017/ Portugal | Randomized pre-test post-test with control | *14* | *13* |  | *18-60 years old* | Down Syndrome | Free run, Heading, Table Tilt, Snowboard Slalom, Tightrope Tension, Hula Hoop, Balance Bubble, Penguin Slide | Wii | 60/3 | 1559/8.66 | Nothing | ⭤ (FBT)  🡑 (TUG) |
| Singh, D. K. A., et al. 2012/ Malaysia | Randomized pre-test post-test with control | *18* | *18* | 0 | *62.6±4.8* | NSC | Ski Slalom, Table Tilt, Penguin Slide, Soccer Heading, Tight Rope Walk, Perfect 10, Tilt City | Wii | 30/2 | 480/6 | CT | 🡑 (TST)  🡑 (TUG) |
| Singh, D. K. A., et al. 2013/ Malaysia | Controlled Trial | *15* | *13* |  | *62.6±4.1* | Stroke | Balance Ball, Rally Bubble | Wii, Xbox 360 Kinect | 30/2 | 360/6 | CT | 🡑 (TUG)  ⭤ (Overall Balance)  ⭤ (BI) |
| Song, J., et al. 2018/  Australia | RCT | *31* | *29* | 40 | *66.6±7* | PD | Stepmania | Computer | 15/3 | 540/12 | Nothing | ⭤ (CSRT Stepping Performance)  ⭤ (CSRT RT)  ⭤ (CSRT MT)  🡑 (TUG) |
| Song, Y. B., et al. 2014/ South Korea | Randomized pre-test post-test with control | *10* | *20* | 53 | *63.4±13.7* | Stroke |  | VR IREX System | 25/5 | 750/3 | Nothing and CT | 🡑 (BBS)  🡑 (FI)  🡑 (SI NO)  ⭤ (SI NC)  🡑 (SI PO)  ⭤ (SI PC)  ⭤ (SI HR)  ⭤ (SI HL) ⭤ (SI HB)  ⭤ (SI HF) |
| Straker, L., et al. 2015/ Australia | Crossover RCT | *10* | *11* | 48 | *11.0±1* | DCD | Sports Champion, Start the Party, TV Superstars, Your Shape Fitness Evolved, Motion Sports, Kinect Adventures, Free Riders, Dance Central, Dr. Kawashima’s Body and Brain Exercises, Racket Sports, Cross Board 7 | Xbox 360, Microsoft Kinect | 20/5 | 1600/16 | Nothing | ⭤ (MABC-2)  ⭤ (Balance Path Distance) |
| Szturm, T., et al. 2011/ Canada* | RCT | *13* | *14* | 30 | *80.8±6.4* | Frail Community-Dwelling Elderly | Under Pressure, Memory Match, Balloon Burst | Computer | 45/2 | 720/8 | CT | 🡑 (BBS)  ⭤ (TUG) |
| Tak, S., et al. 2015/South Korea | Single-blinded RCT | *13* | *13* | 77 | *46.3±9.7* | Spinal Cord Injury | Tennis, Table Tennis, Boxing, Golf, Bowling, Frisbee, Canoe, Sword Play | Wii | 30/3 | 540/6 | Nothing | 🡑 (Sway Distance)  🡑 (Sway Velocity)  🡑 (mFRT) |
| Tarakci, D., et al. 2016/ Turkey | RCT | *19* | *19* | 63 | *10.5±2.74* | CP | Ski Slalom, Tightrope Walk, Soccer Heading | Wii | 20/2 | 1200/12 | CT | 🡑 (TUG)  🡑 (10SCT) |
| Taylor, L., et al. 2018/ Australia* | RCT | *29* | *36* | 26 | *85.3±7.3* | NSC | Your Shape Fitness Evolved, Aging With Grace | Xbox Kinect | 35/2 | 560/8 | CT | ⭤ (TUG) |
| Tollar, J., et al. 2019/ Holland | RCT | *25* | *49* | 49 | *69.4±4.36* | PD | Kinect Adventures | Xbox 360 | 60/5 | 1500/5 | Traditional Exercise, Sedentary Games | 🡑 (BBS)  ⭤ (BESTest)  🡑 (DGI)  🡑 (COP Wide EO)  🡑 (COP Wide EC)  ⭤ (COP Narrow EO)  ⭤ (COP Narrow EC)  ⭤ (COP Tandem EO)  ⭤ (COP Tandem EC) |
| Urgen, M. S., et al. 2016/ Turkey | Randomized pre-test post-test with control | *15* | *15* | 47 | *11.2±2.28* | CP | Wii Fit | Wii | 45/2 | 810/9 | Nothing | 🡑 (TUG)  🡑 (PBS) |
| Uysal, S. A., et al. 2016/ Turkey | Single-blind and randomized | *12* | *12* | 42 | *9.62±2.6* | CP | Wii Sports ( Basketball, Tennis, Boxing) | Wii | 30/2 | 1800/12 | CT | 🡑 (PBS) |
| Van Biljon, A., et al. 2012/South Africa | Randomized pre-test post-test with control | *11* | *20* |  | *11.4* | Overweight and Obese | Aerobic, Boxing, Hula Hoop | Wii | 30/3 | 540/6 | Nothing and Sedentary Games | 🡑 (Balance) |
| van den Berg, M., et al. 2016/ Australia | Randomized Trial | *29* | *29* | 38 | *80±11.5* | Geriatric and Neurological Rehabilitation |  | Xbox Kinect, Wii, Dance Mat, HUMAC balance system, Modular Interactive Stepping Tiles | 60/5 | 3600/12 | CT | ⭤ (RMI) |
| van den Heuvel, M. R. C., et al. 2014/Holland | RCT | *17* | *16* | 61 | *67.5±8.04* | PD | Workstations | LCD Monitor, Computer | 45/2 | 600/5 | CT | ⭤ (BBS)  ⭤ (SLS Preferred Leg)  ⭤ (SLS Non-Preferred Leg) |
| Vernadakis, N., et al. 2012/Greece | Randomized pre-test post-test with control | *16* | *16* | 56 | *20.6±0.62* | NSC | Tree, Standing Knee, King of the Dance, Soccer Heading, Table Tilt, Penguin Slide, Ski Slalom, Tightrope Walk, Snowboard Slalom, Balance Bubble | Wii | 24/2 | 384/8 | CT | 🡑 (SI Right Limp 1)  🡑 (SI Left Limp 2)  🡑 (A/P Index Right Limp 1)  🡑 (A/P Index Left Limp 1) |
| Vernadakis, N., et al. 2018/Greece | Randomized pre-test post-test with control | *10* | *10* | 50 | *18.3±0.7* | Deaf | Wii Fit Plus | Wii | 15/2 | 240/8 | CT | 🡑 (FBT) |
| Whyatt, C., et al. 2015/UK | Randomized pre-test post-test with control | *40* | *42* | 30 | *76.9±6.94* | NSC | Apple Catch, Bubble Pop, Avoid the Shark, Smart Shrimp | Wii | 30/2 | 300/5 | Nothing | 🡑 (BBS) |
| Wuang, Y.-P., et al. 2011/ Taiwan* | Quasi-experimental | *105* | *50* |  | *7-12 years old* | Down Syndrome |  | Wii | 60/2 | 2880/24 | Nothing and CT | 🡑 (BOTMP Balance) |
| Yang, W.-C., et al. 2016/ Taiwan | RCT | *11* | *12* | 61 | *74±7.35* | PD | Basic Learning, Indoor Daily Tasks, Outdoor Daily Tasks | Computer | 50/2 | 600/6 | CT | 🡑 (BBS)  🡑 (DGI)  🡑 (TUG) |
| Yatar, G. I., et al. 2015/ Turkey | RCT | *15* | *15* | 43 | *59.7±13.6* | Stroke | Soccer Heading, Ski Slalom, Balance Bubble | Wii | 30/3 | 720/4 | CT | 🡑 (BBS)  🡑 (TUG)  🡑 (DGI) |
| Yazgan, Y. Z., et al. 2020/ Turkey | RCT | *16* | *31* | 9 | *43.8±9.36* | MS | Penguin Slide, Table Tilt, Ski Slalom, Heading, Balance Bubble | Wii | 60/2 | 960/8 | Nothing | 🡑 (BBS) |
| Yen, C.-Y., et al. 2011/ Taiwan* | RCT | *14* | *28* | 79 | *70.7±6.4* | PD | 3D Ball-rolling | VR Balance Board | 20/2 | 360/6 | Nothing and CT | ⭤ (SOT-1)  ⭤ (SOT-2)  ⭤ (SOT-3)  ⭤ (SOT-4)  ⭤ (SOT-5)  🡑 (SOT-6) |
| Yom, C., et al. 2015/South Korea | Randomized pre-test post-test with control | *10* | *10* | 55 | *71.4* | Stroke | VR-Based Ankle Exercise | Computer, Beam Projector, Screens | 30/5 | 900/6 | CT | 🡑 (TUG) |
| Yoo, H.-N., et al. 2013/South Korea | Randomized pre-test post-test with control | *10* | *11* | 0 | *74.3±4.49* | NSC |  | Computer | 40/3 | 1440/12 | CT | 🡑 (BBS) |
| Yu, J. H., et al. 2016/South Korea | Randomized pre-test post-test with control | *10* | *10* | 60 | *63.8±5.4* | Stroke | Balance Bubble, Ski Slalom, Ski Jump, Soccer Heading, Table Tilting, Penguin Slide | Wii | 30/3 | 540/6 | Nothing | 🡑 (BBS)  🡑 (TUG) |
| Yu, T.-C., et al. 2020/ Taiwan | Randomized pre-test post-test with control | *20* | *20* | 20 | *64.0±4.4* | NSC | Boxing, Beach Volleyball, Football, Track and Field, Table Tennis, Bowling, Kinect Adventures | Xbox Kinect | 50/3 | 1500/10 | Nothing | ⭤ (Static Balance)  ⭤ (Dynamic Balance) |

Abbreviations:

10SCT = 10 Stair Climbing Test

A/P = Anterior/Posterior or Anterior-Posterior

AD = Alzheimer’s Disease

ANT = anterior

BBS = Berg Balance Scale

BBT = Berg Balance Test

BESTest = Balance Evaluation Systems Test

BOTMP = Bruininks-Oseretsky Test of Motor Proficiency

BST = Backwards Stepping Test

C = Control

COPD = Chronic Obstructive Pulmonary Disease

CP = Cerebral Palsy

CSRT = Choice Stepping Reaction Time

CST = Crossover Stepping Test

CT = Conventional Treatment

CTSIB = Clinical Test for Sensory Interaction in Balance

DCD = Developmental Condition Disorder

DD = Developmental Delay

DGI = Dynamic Gait Index

DPSI = Dynamic Postural Stability Index

DTm = Dual task using motor interference

E = Experimental

EC = Eyes Closed

EO = Eyes Open

FBT = Flamingo Balance Test

FGA = Functional Gait Assessment

FRT = Functional Reach Test

FSST = Four Square Step Test

HB = Standing with eyes closed with neck extension

HF = Standing with eyes closed with neck flexion

HL = Standing with eyes closed with head rotation to the left side

HR = Standing with eyes closed with head rotation to the right side

LOS = Limits of Stability

MABC-2 = Movement Assessment Battery for Children Second Edition

MCI = Mild Cognitive Impairment

mFRT = modified Functional Reach Test

ML = Mediolateral/Medial-lateral

MT = Movement Time

NC = Standing with eyes closed

NO = Standing with eyes open

NSC = No Specific Condition

NT = Normal Task

OLB = Overall Balance

OLS = One-leg Stance

OSI = Overall Stability Index

PASS = Postural Assessment Scale for Stroke Patients

PBS = Pediatric Balance Scale

PC = Standing on pillow with eyes closed

PD = Parkinson’s Disease

PL = posterior-lateral

PM = posterior-medial

PO = Standing on pillow with eyes open

PS = Postural Sway

PSC = Postural Stability Control

PSV = Postural Sway Velocity

Resp = Response time

RMI = Rivermead Mobility Index

RT = Reaction Time

SBI = Static Balance Index

SEBT = Star Excursion Balance Test

SI = Stability Index

SLS = Single Leg Standing

SLT = Single-Leg Test

SPPB = Short Physical Performance Battery

sROM = sharpened Romberg’s test

STOLL = Standing on the Left Leg

STORL = Standing on the Right Leg

TST = Ten Step Test

TT = Tinetti’s Test

TUG = Timed Up and Go

TWS = Total COP Displacement During Weight Shift

VVC = Visual-Vestibular Condition

⭤ (no difference)

🡑 (improved)

🡓 (did not improve)

* = no sufficient data provided, not included in the meta-analysis

**Supplementary References**

39. Adcock M, et al. Effects of an in-home multicomponent exergame training on physical functions, cognition, and brain volume of older adults: a randomized controlled trial*.* Front Med 2020;6:321.

40. AlSaif AA, Alsenany S. Effects of interactive games on motor performance in children with spastic cerebral palsy*.* J Phys Ther Sci 2015;27:2001-3.

41. Anson E, et al. Trunk motion visual feedback during walking improves dynamic balance in older adults: Assessor blinded randomized controlled trial*.* Gait Posture 2018;62:342-8.

42. Bang YS, Son KH, Kim HJ. Effects of virtual reality training using Nintendo Wii and treadmill walking exercise on balance and walking for stroke patients. J Phys Ther Sci 2016;28:3112-5.

43. Barcala L, et al. Visual biofeedback balance training using wii fit after stroke: a randomized controlled trial*.* J Phys Ther Sci 2013;25:1027-32.

44. Barry G, et al. Exergaming (XBOX Kinect™) versus traditional gym-based exercise for postural control, flow and technology acceptance in healthy adults: a randomised controlled trial. BMC Sports Sci Med Rehabil 2016;8:25.

45. Cai, H, et al. Effect of low-intensity, Kinect™-Based Kaimai-Style Qigong exercise in older adults with type 2 diabetes*.* J Gerontol Nurs 2019;45:42-52.

46. Chao Y-Y, et al. Physical and psychosocial effects of Wii Fit exergames use in assisted living residents: a pilot study*.* Clin Nurs Res 2015;24:589-603.

47. Chen CL, et al. Muscle strength enhancement following home-based virtual cycling training in ambulatory children with cerebral palsy*.* Res Dev Disabil 2012;33:1087-94.

48. Cho GH, Hwangbo G, Shin HS. The effects of virtual reality-based balance training on balance of the elderly*.* J Phys Ther Sci 2014;26:615-7.

49. Cho H, Sohng KY. The effect of a virtual reality exercise program on physical fitness, body composition, and fatigue in hemodialysis patients*.* J Phys Ther Sci 2014;26:1661-5.

50. Cho KH, Lee KJ, Song CH. Virtual-reality balance training with a video-game system improves dynamic balance in chronic stroke patients*.* Tohoku J Exp Med 2012;228:69-74.

51. Choi D, Choi W, Lee S. Influence of Nintendo Wii fit balance game on visual perception, postural balance, and walking in stroke survivors: a pilot randomized clinical trial*.* Games Health 2018;7:377-84.

52. Choi W, Lee S. The effects of virtual Kayak Paddling exercise on postural balance, muscle performance, and cognitive function in older adults with mild cognitive impairment: a randomized controlled trial*.* J Aging Phys Act 2019;27:861-70.

53. Chow DH, Mann SK. Effect of cyber-golfing on balance amongst the elderly in Hong Kong: a pilot randomised trial*.* Hong Kong J Occup Ther 2015;26:9-13.

54. Cikajlo I, et al. Multi-exergames to set targets and supplement the intensified conventional balance training in patients with stroke: a randomized pilot trial. Front Psychol 2020;11:572.

55. Cimino V, Chisari C, Zagari F. Effects of Nintendo Wii Fit® balance exercise program on physical abilities and quality of life in multiple sclerosis patients*.* J Neurol Neurorehabil Res 2019;4:1-7.

56. da Fonseca EP, da Silva NMR, Pinto EB. Therapeutic effect of virtual reality on post-stroke patients: randomized clinical trial*.* J Stroke Cerebrovasc Dis 2017;26:94-100.

57. Ditchburn J-L, et al. The effects of exergaming on pain, postural control, technology acceptance and flow experience, in older people with chronic musculoskeletal pain: a randomised controlled trial. BMC Sports Sci Med Rehabil 2020;12:63.

58. Duque, G, et al. Effects of balance training using a virtual-reality system in older fallers. Clin Interv Aging 2013;8:257-63.

59. Eftekharsadat B, et al. Effect of virtual reality-based balance training in multiple sclerosis*.* Neurol Res 2015;37:539-44.

60. Fakhro MA, Hadchiti R, Awad B. Effects of Nintendo Wii fit game training on balance among Lebanese older adults. Aging Clin Exp Res 2019:1-8.

61. Feng H, et al. Virtual reality rehabilitation versus conventional physical therapy for improving Balance and Gait in Parkinson’s disease patients: a randomized controlled trial. Med Sci Monit 2019;25:4186-92.

62. Ferguson GD, et al. The efficacy of two task-orientated interventions for children with developmental coordination disorder: Neuromotor Task Training and Nintendo Wii Fit Training. Res Dev Disabil 2013;34:2449-61.

63. Fitzgerald D, et al. Effects of a wobble board-based therapeutic exergaming system for balance training on dynamic postural stability and intrinsic motivation levels*.* J Orthop Sports Phys Ther 2010;40:11-9.

64. Franco JR, et al. The effect of the Nintendo Wii Fit and exercise in improving balance and quality of life in community dwelling elders*.* Technol Health Care 2012;20:95-115.

65. Fritz SL, et al. Active video-gaming effects on balance and mobility in individuals with chronic stroke: a randomized controlled trial*.* Top Stroke Rehabil 2013;20:218-25.

66. Gandolfi M, et al. Virtual reality telerehabilitation for postural instability in Parkinson’s disease: a multicenter, single-blind, randomized, controlled trial. BioMed Res Int 2017;2017:7962826.

67. Gatica-Rojas V, et al. Does Nintendo Wii Balance Board improve standing balance? A randomized controlled trial in children with cerebral palsy. Eur J Phys Rehabil Med 2017;53:535-44.

68. Gioftsidou A, et al. Typical balance exercises or exergames for balance improvement? J Back Musculoskelet Rehabil 2013;26:299-305.

69. Gutierrez RO, et al. A telerehabilitation program by virtual reality-video games improves balance and postural control in multiple sclerosis patients. NeuroRehabilitation 2013;33:545-54.

70. Hamari L, et al. The effect of an active video game intervention on physical activity, motor performance, and fatigue in children with cancer: a randomized controlled trial. BMC Res Notes 2019;12:784.

71. Henrique PPB, Colussi EL, De Marchi ACB. Effects of exergame on patients’ balance and upper limb motor function after stroke: a randomized controlled trial. J Stroke Cerebrovasc Dis 2019;28:2351-7.

72. Huang H-C, et al. Can using exergames improve physical fitness? A 12-week randomized controlled trial. Comput Human Behav 2017;70:310-6.

73. Hung ES, et al. Effects of interactive video game-based exercise on balance in diabetic patients with peripheral neuropathy: an open-level, crossover pilot study. Evid Based Complement Alternat Med 2019;2019:4540709.

74. Hung J-W, et al. Randomized comparison trial of balance training by using exergaming and conventional weight-shift therapy in patients with chronic stroke. Arch Phys Med Rehabil 2014;95:1629-37.

75. Hung J-W, et al. Feasibility of using tetrax biofeedback video games for balance training in patients with chronic hemiplegic stroke. PM R 2016;8:962-70.

76. Ibrahim MS, Mattar AG, Elhafez SM. Efficacy of virtual reality-based balance training versus the Biodex balance system training on the body balance of adults. J Phys Ther Sci 2016;28:20-6.

77. Jorgensen MG, et al. Efficacy of Nintendo Wii training on mechanical leg muscle function and postural balance in community-dwelling older adults: a randomized controlled trial. J Gerontol A Biol Sci Med Sci 2013;68:845-52.

78. Ju Y-J, et al. The effect of laboratory-developed video games on balance performance in children with developmental coordination disorder. Biomed Eng Appl Basis Commun 2018;30:1850005.

79. Jung J, Yu J, Kang H. Effects of virtual reality treadmill training on balance and balance self-efficacy in stroke patients with a history of falling. J Phys Ther Sci 2012;24:1133-6.

80. Kalron A, et al. The effect of balance training on postural control in people with multiple sclerosis using the CAREN virtual reality system: a pilot randomized controlled trial. J Neuroeng Rehabil 2016;13:13.

81. Kannan L, et al. Cognitive-motor exergaming for reducing fall risk in people with chronic stroke: a randomized controlled trial. NeuroRehabilitation 2019;44:493-510.

82. Karahan AY, et al. Effects of exergames on balance, functional mobility, and quality of life of geriatrics versus home exercise programme: randomized controlled study. Cent Eur J Public Health 2015;23 Suppl(Supplement):S14-8.

83. Karasu AU, Batur EB, Karataş GK. Effectiveness of Wii-based rehabilitation in stroke: a randomized controlled study. J Rehabil Med 2018;50:406-12.

84. Karssemeijer EGA, et al. Exergaming as a physical exercise strategy reduces frailty in people with dementia: a randomized controlled trial. J Am Med Dir Assoc 2019;20:1502-8.

85. Katajapuu N, et al. Benefits of exergame exercise on physical functioning of elderly people. In: 8th IEEE International Conference on Cognitive Infocommunications (CogInfoCom). 2017. IEEE.

86. Khurana M, Walia S, Noohu MM. Study on the effectiveness of virtual reality game-based training on balance and functional performance in individuals with paraplegia. Top Spinal Cord Inj Rehabil 2017;23:263-70.

87. Khushnood K, et al. Does Wii Fit balance training improve balance and reduce fall risk in diabetic patients as compared to balance training exercises? A randomized control trial. Rawal Med J 2019;44:44-48.

88. Kim J, et al. Unsupervised virtual reality-based exercise program improves hip muscle strength and balance control in older adults: a pilot study. Arch Phys Med Rehabil 2013;94:937-43.

89. Kliem A, Wiemeyer J. Comparison of a traditional and a video game based balance training program. Int J Comput Sci Sport 2010;9:80-91.

90. Kramer A, Dettmers C, Gruber M. Exergaming with additional postural demands improves balance and gait in patients with multiple sclerosis as much as conventional balance training and leads to high adherence to home-based balance training. Arch Phys Med Rehabil 2014;95:1803-9.

91. Kwok BC, Pua YH. Effects of WiiActive exercises on fear of falling and functional outcomes in community-dwelling older adults: a randomised control trial. Age Ageing 2016;45:621-7.

92. Lai CH, et al. Effects of interactive video-game based system exercise on the balance of the elderly. Gait Posture 2013;37:511-5.

93. Laver K, et al. Use of an interactive video gaming program compared with conventional physiotherapy for hospitalised older adults: a feasibility trial. Disabil Rehabil 2012;34:1802-8.

94. Lee C-H, Kim Y, Lee B-H. Augmented reality-based postural control training improves gait function in patients with stroke: randomized controlled trial. Hong Kong Physiother J 2014;32:51-7.

95. Lee H-C, et al. The effect of a virtual reality game intervention on balance for patients with stroke: a randomized controlled trial. Games Health 2017;6:303-11.

96. Lee HY, Kim YL, Lee SM. Effects of virtual reality-based training and task-oriented training on balance performance in stroke patients. J Phys Ther Sci 2015;27:1883-8.

97. Lee IW, Kim YN, Lee DK. Effect of a virtual reality exercise program accompanied by cognitive tasks on the balance and gait of stroke patients. J Phys Ther Sci 2015;27:2175-7.

98. Lee NY, Lee DK, Song HS. Effect of virtual reality dance exercise on the balance, activities of daily living, and depressive disorder status of Parkinson’s disease patients. J Phys Ther Sci 2015;27:145-7.

99. Lee S, Shin S. Effectiveness of virtual reality using video gaming technology in elderly adults with diabetes mellitus. Diabetes Technol Ther 2013;15:489-96.

100. Lee Y, et al. Virtual reality training with three-dimensional video games improves postural balance and lower extremity strength in community-dwelling older adults. J Aging Phys Activity 2017;25:621-7.

101. Leutwyler H, et al. Impact of a pilot videogame-based physical activity program on walking speed in adults with schizophrenia. Community Ment Health J 2018;54:735-9.

102. Liao Y-Y, et al. Effects of virtual reality-based physical and cognitive training on executive function and dual-task gait performance in older adults with mild cognitive impairment: a randomized control trial. Front Aging Neurosci 2019;11:162.

103. Liao Y-Y, Chen IH, Wang R-Y. Effects of Kinect-based exergaming on frailty status and physical performance in prefrail and frail elderly: a randomized controlled trial. Sci Rep 2019;9:9353.

104. Liao YY, et al. Virtual reality-based training to improve obstacle-crossing performance and dynamic balance in patients with Parkinson’s disease. Neurorehabil Neural Repair 2015;29:658-67.

105. Lin YT, Lee WC, Hsieh RL. Active video games for knee osteoarthritis improve mobility but not WOMAC score: a randomized controlled trial. Ann Phys Rehabil Med 2020;63:458-65.

106. Lloréns R, et al. Improvement in balance using a virtual reality-based stepping exercise: a randomized controlled trial involving individuals with chronic stroke. Clin Rehabil 2015;29:261-8.

107. Martín-Martínez JP, et al. Effects of 24-week exergame intervention on physical function under single-and dual-task conditions in fibromyalgia: a randomized controlled trial. Scand J Med Sci Sports 2019;29:1610-7.

108. McEwen D, et al. Virtual reality exercise improves mobility after stroke: an inpatient randomized controlled trial. Stroke 2014;45:1853-5.

109. Mombarg R, Jelsma D, Hartman E. Effect of Wii-intervention on balance of children with poor motor performance. Res Dev Disabil 2013;34:2996-3003.

110. Montero-Alía P, et al. Controlled trial of balance training using a video game console in community-dwelling older adults. Age Ageing 2019;48:506-12.

111. Morat M, et al. Effects of stepping exergames under stable versus unstable conditions on balance and strength in healthy community-dwelling older adults: a three-armed randomized controlled trial. Exp Gerontol 2019;127:110719.

112. Morone G, et al. Wii Fit is effective in women with bone loss condition associated with balance disorders: a randomized controlled trial. Aging Clin Exp Res 2016;28:1187-93.

113. Morone G, et al. The efficacy of balance training with video game-based therapy in subacute stroke patients: a randomized controlled trial. Biomed Res Int 2014;2014:580861.

114. Morrison S, et al. Supervised balance training and Wii Fit-based exercises lower falls risk in older adults with type 2 diabetes. J Am Med Dir Assoc 2018;19:185.e7-13.

115. Mugueta-Aguinaga I, Garcia-Zapirain B. FRED: exergame to prevent dependence and functional deterioration associated with ageing. A pilot three-week randomized controlled clinical trial. Int J Environ Res Public Health 2017;14:1439.

116. Nicholson VP, et al. Six weeks of unsupervised Nintendo Wii Fit gaming is effective at improving balance in independent older adults. J Aging Phys Activity 2015;23:153-8.

117. Nilsagård YE, Forsberg AS, von Koch L. Balance exercise for persons with multiple sclerosis using Wii games: a randomised, controlled multi-centre study. Mult Scler J 2013;19:209-16.

118. Ordnung M, et al. No overt effects of a 6-week exergame training on sensorimotor and cognitive function in older adults. A preliminary investigation. Front Human Neurosci 2017;11:160.

119. Padala KP, et al. Home-based exercise program improves balance and fear of falling in community-dwelling older adults with mild Alzheimer’s disease: a pilot study. J Alzheimers Dis 2017;59:565-74.

120. Padala KP, et al. Wii-fit for improving gait and balance in an assisted living facility: a pilot study. J Aging Res 2012;2012:597573.

121. Park EC, Kim SG, Lee CW. The effects of virtual reality game exercise on balance and gait of the elderly. J Phys Ther Sci 2015;27:1157-9.

122. Park J, Yim J. A new approach to improve cognition, muscle strength, and postural balance in community-dwelling elderly with a 3-D virtual reality Kayak Program. Tohoku J Exp Med 2016;238:1-8.

123. Pedreira da Fonseca E, da Silva Ribeiro NM, Pinto EB. Therapeutic effect of virtual reality on post-stroke patients: randomized clinical trial. J Stroke Cerebrovasc Dis 2017;26:94-100.

124. Pompeu J, et al. Safety, feasibility and effectiveness of balance and gait training using Nintendo Wii Fit Plus on unstable surface in patients with Parkinson’s disease: a pilot study. J Alzheimers Dis Parkinsonism 2014;4:196-204.

125. Portela FR, et al. Wiitherapy on seniors—effects on physical and metal domains. In: 2011 IEEE 1st International Conference on Serious Games and Applications for Health (SeGAH). 2011. IEEE.

126. Prosperini L, et al. Home-based balance training using the Wii balance board a randomized, crossover pilot study in multiple sclerosis. Neurorehabil Neural Repair 2013;27:516-25.

127. Ribas CG, et al. Effectiveness of exergaming in improving functional balance, fatigue and quality of life in Parkinson’s disease: a pilot randomized controlled trial. Parkinsonism Relat Disord 2017;38:13-8.

128. Rica RL, et al. Effects of a Kinect-based physical training program on body composition, functional fitness and depression in institutionalized older adults. Geriatr Gerontol Int 2020;20:195-200.

129. Rosiak O, et al. Evaluation of the effectiveness of a virtual reality-based exercise program for unilateral peripheral vestibular deficit. J Vestib Res 2018;28:409-15.

130. Rutkowski S, et al. Virtual reality rehabilitation in patients with chronic obstructive pulmonary disease: a randomized controlled tial. Int J Chronic Obstruct Pulm Dis 2020;15:117-24.

131. Şahin S, et al. The effects of virtual reality on motor functions and daily life activities in unilateral spastic cerebral palsy: a single-blind randomized controlled trial. Games Health J 2020;9:45-52.

132. Salem Y, et al. Effectiveness of a low-cost virtual reality system for children with developmental delay: a preliminary randomised single-blind controlled trial. Physiotherapy 2012;98:189-95.

133. Santos P, et al. Efficacy of the Nintendo Wii combination with conventional exercises in the rehabilitation of individuals with Parkinson’s disease: a randomized clinical trial. NeuroRehabilitation 2019;45:255-63.

134. Sato K, et al. Improving walking, muscle strength, and balance in the elderly with an exergame using Kinect: a randomized controlled trial. Games Health J 2015;4:161-7.

135. Schoene D, et al. A randomized controlled pilot study of home-based step training in older people using videogame technology. PLoS One 2013;8:e57734.

136. Sheehan DP, Katz L. The effects of a daily, 6-week exergaming curriculum on balance in fourth grade children. J Sport Health Sci 2013;2:131-7.

137. Shen X, Mak MK. Technology-assisted balance and gait training reduces falls in patients with Parkinson's disease: a randomized controlled trial with 12-month follow-up. Neurorehabil Neural Repair 2015;29:103-11.

138. Shih M-C, et al. Effects of a balance-based exergaming intervention using the Kinect sensor on posture stability in individuals with Parkinson’s disease: a single-blinded randomized controlled trial. J Neuroeng Rehabil 2016;13:78.

139. Silva V, et al. Wii-based exercise program to improve physical fitness, motor proficiency and functional mobility in adults with Down syndrome. J Intellect Disabil Res 2017;61:755-65.

140. Singh DK, et al. Effects of balance-focused interactive games compared to therapeutic balance classes for older women. Climacteric 2013;16:141-6.

141. Singh DKA, et al. Effects of substituting a portion of standard physiotherapy time with virtual reality games among community-dwelling stroke survivors. BMC Neurol 2013;13:199.

142. Song J, et al. Home-based step training using videogame technology in people with Parkinson’s disease: a single-blinded randomised controlled trial. Clin Rehabil 2018;32:299-311.

143. Song YB, et al. The effect of virtual reality and tetra-ataxiometric posturography programs on stroke patients with impaired standing balance. Ann Rehabil Med 2014;38:160-6.

144. Straker L, et al. A crossover randomised and controlled trial of the impact of active video games on motor coordination and perceptions of physical ability in children at risk of developmental coordination disorder. Hum Mov Sci 2015;42:146-60.

145. Szturm T, et al. Effects of an interactive computer game exercise regimen on balance impairment in frail community-dwelling older adults: a randomized controlled trial. Phys Ther 2011;91:1449-62.

146. Tak S, Choi W, Lee S. Game-based virtual reality training improves sitting balance after spinal cord injury: a single-blinded, randomized controlled trial. Med Sci Monit 2015;56:53-9.

147. Tarakci D, et al. Effects of Nintendo Wii-Fit((R)) video games on balance in children with mild cerebral palsy. Pediatr Int 2016;58:1042-50.

148. Taylor L, et al. Exergames to improve the mobility of long-term care residents: a cluster randomized controlled trial. Games Health 2018;7:37-42.

149. Tollar J, Nagy F, Hortobagyi T. Vastly different exercise programs similarly improve Parkinsonian symptoms: a randomized clinical trial. Gerontology 2019;65:120-7.

150. Ürgen MS, et al. Investigation of the effects of the Nintendo® Wii-Fit training on balance and advanced motor performance in children with spastic hemiplegic cerebral palsy: a randomized controlled trial. Int J Ther Rehabil Res 2016;5:146.

151. Uysal SA, Baltaci G. Effects of Nintendo Wii™ training on occupational performance, balance, and daily living activities in children with spastic hemiplegic cerebral palsy: a single-blind and randomized trial. Games Health J 2016;5:311-7.

152. Van Biljon A, Longhurst G. The influence of exergaming on the functional fitness in overweight and obese children:: physical activity, health and wellness. Afr J Phys Health Educ Rec Dance 2012;18(Issue-4_2):984-91.

153. van den Berg M, et al. Video and computer-based interactive exercises are safe and improve task-specific balance in geriatric and neurological rehabilitation: a randomised trial. J Physiother 2016;62:20-8.

154. van den Heuvel MR, et al. Effects of augmented visual feedback during balance training in Parkinson's disease: a pilot randomized clinical trial. Parkinsonism Relat Disord 2014;20:1352-8.

155. Vernadakis N, et al. The impact of Nintendo Wii to physical education students’ balance compared to the traditional approaches. Comput Educ 2012;59:196-205.

156. Vernadakis N, et al. The effect of an exergame-based intervention on balance ability on deaf adolescents. Sport Sci 2018;11:36-41.

157. Whyatt C, et al. A Wii Bit of Fun: a novel platform to deliver effective balance training to older adults. Games Health J 2015;4:423-33.

158. Wuang Y-P, et al. Effectiveness of virtual reality using Wii gaming technology in children with Down syndrome. Res Dev Disabil 2011;32:312-321.

159. Yang WC, et al. Home-based virtual reality balance training and conventional balance training in Parkinson’s disease: a randomized controlled trial. J Formos Med Assoc 2016;115:734-43.

160. Yatar GI, Yildirim SA. Wii Fit balance training or progressive balance training in patients with chronic stroke: a randomised controlled trial. J Phys Ther Sci 2015;27:1145-51.

161. Yazgan YZ, et al. Comparison of the effects of two different exergaming systems on balance, functionality, fatigue, and quality of life in people with multiple sclerosis: a randomized controlled trial. Mult Scler Relat Disord 2020;39:101902.

162. Yen CY, et al. Effects of virtual reality-augmented balance training on sensory organization and attentional demand for postural control in people with Parkinson disease: a randomized controlled trial. Phys Ther 2011;91:862-74.

163. Yom C, Cho HY, Lee B. Effects of virtual reality-based ankle exercise on the dynamic balance, muscle tone, and gait of stroke patients. J Phys Ther Sci 2015;27:845-9.

164. Yoo HN, Chung E, Lee BH. The effects of augmented reality-based Otago exercise on balance, gait, and falls efficacy of elderly women. J Phys Ther Sci 2013;25:797-801.

165. Yu JH, Cho KH. Effectiveness of virtual reality game on functional movement and activities of daily living in hemiparetic stroke patients. J Nanoelectron Optoelectron 2016;11:98-102.

166. Yu TC, et al. Effects of exergames on physical fitness in middle-aged and older adults in Taiwan. Int J Environ Res Public Health 2020;17:2565.
